# Genome-Wide Identification and Characterization of the TBL Gene Family and Temporal Expression Dynamics During Powdery Mildew Infection in Cucumber (*Cucumis sativus*)

**DOI:** 10.3390/biology15171454

**Published:** 2026-08-25

**Authors:** Wenxuan Chu, Zixuan Li, Yihe Tian, Ziyi Zhang, Ruigang Wu

**Affiliations:** School of Landscape and Ecological Engineering, Hebei University of Engineering, Handan 056038, China

**Keywords:** TBL gene family, cell-wall polysaccharides, O-acetylation, temporal expression dynamics, powdery mildew

## Abstract

Cell-wall polysaccharide O-acetylation contributes to cell-wall assembly, organ development, and plant–pathogen interactions, but the cucumber TBL gene family remains poorly characterized. Here, 37 *CsTBL* genes were identified genome-wide and analyzed using phylogenetic, syntenic, conserved-motif, gene-structure, promoter, protein-structure, Gene Ontology, and transcriptome approaches, followed by RT-qPCR analysis after powdery mildew inoculation. All CsTBL proteins contained the conserved GDS and DxxH motifs, whereas accessory motifs and predicted structural features varied among clades. Intraspecific analysis identified dispersed, WGD/segmental, and tandem duplication categories, and cross-species synteny was more extensive with melon than with Arabidopsis. Homology-derived annotations associated *CsTBL* genes with cell-wall polysaccharide metabolism, Golgi/endomembrane compartments, and O-acetyltransferase activity, including six genes assigned to xylan O-acetyltransferase-related annotations. Expression profiling revealed tissue- and developmental-stage-dependent patterns, whereas the publicly available powdery mildew RNA-seq dataset provided descriptive temporal expression profiles in *Podosphaera xanthii*-inoculated samples. Independent RT-qPCR analysis using time-matched mock controls revealed distinct post-inoculation responses among six selected genes. Relative to the corresponding mock controls, *CsTBL2* was consistently repressed; *CsTBL15* showed transient induction at 1 dpi followed by repression; *CsTBL24* exhibited a biphasic response; *CsTBL25* was induced at all sampled post-inoculation time points; *CsTBL26* showed progressive induction; and *CsTBL30* reached its highest observed expression level at 3 dpi. Integrated functional annotation and expression evidence highlighted CsTBL26 as a priority candidate for further functional characterization, while CsTBL24 and CsTBL25 represented fruit-associated candidates with distinct powdery mildew responses; CsTBL30 remained an additional strongly infection-responsive candidate. These findings provide an evolutionary and expression-based framework for the functional characterization of the cucumber TBL gene family.

## Simple Summary

Cucumber fruit growth and resistance to disease depend in part on changes in the plant cell wall, but the genes involved in these processes are not fully understood. In this study, we examined a family of 37 cucumber genes that are predicted to help modify sugars in the cell wall. We compared their sequences, chromosome locations, evolutionary relationships, predicted regulatory features, and expression patterns in different tissues and under several stress conditions. We also experimentally measured the responses of six selected genes during powdery mildew infection, a common fungal disease of cucumber. The genes showed diverse expression patterns, with some being more active in fruit-related tissues and others changing their activity after infection. In particular, CsTBL26 showed a progressive increase in expression during infection and was predicted to be associated with a chemical modification of cell-wall sugars, making it an attractive candidate for future functional studies. These findings provide a useful resource for future research on cucumber fruit quality, storage performance, and disease resistance, although direct effects on these agricultural traits still require experimental validation.

## 1. Introduction

The plant cell wall is a dynamic structure that maintains cell shape, provides mechanical strength to tissues, and mediates intercellular interactions. Its polysaccharide composition and chemical modifications are continuously adjusted in response to organ development and environmental changes. O-acetylation is an important modification of non-cellulosic polysaccharides, including xylan, xyloglucan, mannan, and pectin, and can influence polysaccharide conformation, interactions between cell-wall polymers, and the physicochemical properties of the wall. TRICHOME BIREFRINGENCE-LIKE (TBL) proteins belong to the plant-specific DUF231 family and were initially identified through the abnormal cellulose deposition observed in the trichomes of the *Arabidopsis thaliana* mutant. Subsequent studies demonstrated that many TBL proteins function as polysaccharide O-acetyltransferases associated with the Golgi endomembrane system and typically contain conserved GDS and DxxH sequence motifs [1,2]. Individual TBL members exhibit distinct substrate specificities. AXY4/TBL27 and AXY4L participate in xyloglucan O-acetylation, TBL23–TBL26 catalyze mannan acetylation, TBL10 is involved in the acetylation of pectic rhamnogalacturonan-I, whereas ESK1/TBL29, TBL3, and TBL31–TBL35 contribute to regioselective xylan acetylation [3,4,5,6]. Structural and biochemical characterization of XOAT1/TBL29 further demonstrated that its catalytic center, comprising Ser, Asp, and His residues, forms a covalent acyl-enzyme intermediate, providing direct evidence that the conserved motifs of TBL proteins are catalytically important.

TBL-mediated cell-wall modification is important not only for cell-wall assembly and plant growth but also for environmental adaptation and may influence plant responses during pathogen infection. The *Arabidopsis pmr5/tbl44* mutant exhibits both altered cell-wall composition and enhanced resistance to powdery mildew. By contrast, disruption of *OsTBL1* and *OsTBL2* in rice reduces xylan acetylation, restricts plant growth, and increases susceptibility to bacterial blight. These contrasting phenotypes suggest that the effects of TBL activity on plant immunity may depend on the polysaccharide substrate modified, the tissue context, and the pathogen involved [7,8]. In recent years, genome-wide analyses of the TBL family have been conducted in species including rose, cotton, and eucalyptus. These studies have revealed patterns of gene duplication and tissue-specific expression, identified family members responsive to *Botrytis cinerea* infection or low-temperature stress, and functionally validated several candidate genes [9,10]. Nevertheless, previous research has largely focused either on the functions of individual genes in model plants or on family-wide characterization within a single species. In horticultural crops, systematic evidence remains limited regarding how TBL family members have been retained, expanded, and functionally differentiated across different evolutionary scales, and how these evolutionary patterns are associated with fruit development and disease responses.

Cucumber (*Cucumis sativus* L.) is an economically important fruit vegetable whose fruit development involves rapid cell division, cell expansion, and extensive cell-wall remodeling. Transcriptomic studies have shown that expression modules associated with the cell wall and cytoskeleton are enriched during the rapid fruit expansion stage, suggesting that cell-wall polysaccharide metabolism is an important determinant of fruit tissue development [11,12]. The cucumber genome lacks a recent whole-genome duplication and has undergone extensive chromosome fusion and rearrangement since its divergence from melon (*Cucumis melo*). Cucumber has seven chromosomes, whereas the closely related melon has 12, although large syntenic regions remain conserved between the two genomes [11,13,14]. Consequently, analysis of the TBL family within the cucumber genome alone cannot readily distinguish ancient conserved members from duplicates specific to the cucumber lineage. The functionally well-annotated *Arabidopsis* genome provides a suitable distantly related dicot reference through which experimentally characterized *AtTBL* members can be used to infer potential substrate preferences and functional clades. Melon, in contrast, provides a closely related cucurbit reference for examining gene retention, gene loss, and changes in synteny following chromosome rearrangements. This dual-scale strategy, combining a distantly related functional reference with a closely related evolutionary reference, can improve the reliability of functional predictions for TBL candidates while avoiding the assignment of functions solely on the basis of sequence similarity.

To our knowledge, the cucumber TBL family has not yet been investigated using an integrated framework combining systematic identification, cross-species synteny, fruit-tissue expression, expression patterns in public biotic- and abiotic-treatment datasets, and experimental characterization of *CsTBL* transcriptional dynamics across a powdery mildew infection time course. In this study, we performed genome-wide identification of the cucumber TBL family and systematically analyzed the physicochemical properties, conserved structural features, phylogenetic relationships, gene structures, promoter cis-regulatory elements, chromosomal distributions, and duplication modes of its members. Using *Arabidopsis* and melon as references, we further examined the cross-species syntenic relationships of *CsTBL* genes at both distant and close evolutionary scales. Transcriptomic datasets from different tissues and developmental stages and under multiple biotic and abiotic stresses were integrated to identify candidate genes potentially involved in fruit cell-wall development and environmental responses. Their temporal expression patterns during powdery mildew infection were subsequently examined by RT-qPCR. This study aims to establish an evolutionary and expression framework for the cucumber TBL family and to provide candidate genes and a theoretical basis for further elucidating *CsTBL*-mediated cell-wall polysaccharide acetylation and its potential roles in fruit quality development and disease responses.

## 2. Materials and Methods

### 2.1. Identification and Screening of TBL Gene Family Members in Cucumber

The cucumber genome sequence and gene annotation files were downloaded from the Cucumber Multi-omics Database (http://www.cucumberdb.com/, accessed on 10 March 2026) [15]. Protein sequences of the TBL gene family in the model plant, *Arabidopsis thaliana*, were obtained from The Arabidopsis Information Resource (TAIR; https://www.arabidopsis.org/, accessed on 10 March 2026) and used as reference sequences. Based on the GFF3 annotation file, the longest transcript of each cucumber gene was retained [16]. The corresponding protein sequences were then searched against the cucumber proteome using the BLASTP module implemented in TBtools-II [17]. The E-value threshold was set to 1 × 10^−10^, and only alignments with sequence coverage of at least 50% were retained to obtain an initial set of candidate proteins.

Hidden Markov model (HMM) profiles corresponding to the PF13839 and PF14416 domains were subsequently downloaded from the Pfam database (http://pfam.xfam.org/, accessed on 10 March 2026) [9,18]. The HMM Search function in TBtools was used to identify all putative TBL proteins in the cucumber proteome, with an E-value threshold of 1 × 10^−5^ [17]. The BLASTP- and HMM-derived candidate sets were combined, and redundant protein sequences were removed using CD-HIT v4.8.1 [19]. Finally, the domain composition and integrity of the remaining candidate proteins were examined using the SMART web server (https://smart.embl.de/, accessed on 10 March 2026), and proteins lacking conserved TBL domains were excluded.

### 2.2. Physicochemical Property Analysis of TBL Proteins in Cucumber

The full-length amino acid sequences of all cucumber TBL family members identified at the genome-wide level were used for physicochemical characterization. Core physicochemical properties were calculated in batch using the Protein Properties Calculation module in TBtools-II [17], including protein length, theoretical molecular weight (MW), theoretical isoelectric point (pI), instability index, aliphatic index, and grand average of hydropathicity (GRAVY). The instability index was calculated using the amino acid dipeptide composition-based method proposed by Guruprasad et al., with a value of 40 used as the empirical threshold for evaluating protein stability [20]. GRAVY values were calculated according to the amino acid hydropathy scale developed by Kyte and Doolittle [21]. After format standardization and inspection for anomalous values, the resulting data were used for subsequent characterization of the cucumber TBL family.

### 2.3. Phylogenetic Tree Construction

Protein sequences of the *Arabidopsis thaliana* TBL family were downloaded from the TAIR database (https://www.arabidopsis.org/, accessed on 10 March 2026) and combined with the cucumber CsTBL protein sequences. Multiple sequence alignment of the full-length protein sequences was performed using MEGA 11, and the phylogenetic tree was reconstructed using the neighbor-joining (NJ) method based on amino acid p-distances [22]. Uniform rates among sites and homogeneous substitution patterns among lineages were assumed. Gaps and missing data were treated using partial deletion with a 50% site-coverage cutoff. Node support was evaluated using 1000 bootstrap replicates, and bootstrap values ≥70% were considered to indicate well-supported relationships, whereas nodes with lower support were interpreted cautiously. The resulting phylogenetic tree was visualized and annotated using the Interactive Tree of Life (iTOL) platform [23].

### 2.4. Conserved Motif, Gene Structure, and Promoter Cis-Regulatory Element Analyses

The full-length protein sequences of all cucumber *CsTBL* family members were submitted to the MEME online server for conserved motif prediction [24], with the maximum number of motifs set to 10. Based on the MEME results and the cucumber genome GFF3 annotation file, the conserved protein motifs and exon–intron structures of *CsTBL* genes were integrated and visualized using TBtools to generate a schematic representation of the *CsTBL* gene family structure [17].

For promoter analysis, the 2000-bp genomic sequence upstream of the start codon (ATG) of each *CsTBL* gene was extracted from the cucumber genome using TBtools and defined as the putative promoter region. Cis-regulatory elements within these promoter sequences were identified using PlantCARE (http://bioinformatics.psb.ugent.be/webtools/plantcare/html/, accessed on 10 March 2026), and the results were visualized using TBtools [25].

### 2.5. Secondary and Tertiary Structure Analyses of CsTBL Proteins

The amino acid sequences of the identified cucumber *CsTBL* family members were submitted to the SOPMA tool on the NPSA online platform (https://npsa.lyon.inserm.fr/cgi-bin/npsa_automat.pl?page=/NPSA/npsa_sopma.html, accessed on 10 March 2026) for secondary structure prediction [26]. Based on the prediction results, the proportions of α-helices, extended strands, β-turns, and random coils in each CsTBL protein were calculated and compared to evaluate variation in secondary structure composition among family members.

The three-dimensional structures of cucumber CsTBL proteins were subsequently predicted by homology modeling using the SWISS-MODEL interactive workspace (https://www.swissmodel.expasy.org/, accessed on 10 March 2026) [27]. The GMQE and QMEAN-related quality estimates provided by SWISS-MODEL were inspected during model evaluation. The resulting models were used only for qualitative comparison of predicted structural features. The predicted protein structures were visualized using PyMOL (Version 3.1.8; Schrödinger, LLC, New York, NY, USA).

### 2.6. Chromosomal Localization and Collinearity Analyses

The chromosome numbers, genomic start and end coordinates, and strand orientations of all *CsTBL* genes were extracted from the cucumber genome annotation GFF file. Their physical positions on cucumber chromosomes were visualized using TBtools-II (v2.322) [17].

An all-against-all BLASTP search was performed using the complete cucumber proteome, with an E-value threshold of 1 × 10^−5^. The resulting protein similarity data were combined with chromosome-position information and analyzed using the MCScanX algorithm implemented in TBtools to identify duplicated gene pairs involving *CsTBL* genes and to distinguish tandem duplication from segmental duplication events [28]. Chromosomal distributions and intragenomic collinear relationships of the *CsTBL* genes were visualized using the Advanced Circos module in TBtools.

### 2.7. Expression Analysis of CsTBL Genes

Publicly available transcriptomic data for cucumber cultivar 9930 were obtained from the Cucumber Multi-omics Database. The corresponding raw sequencing datasets and associated experimental metadata are publicly available in the Genome Sequence Archive (GSA) of the National Genomics Data Center, China National Center for Bioinformation, under accession CRA016401 (BioProject PRJCA025716) [15]. The datasets used in the present study included transcriptomic profiles from various tissues and developmental stages under normal growth conditions, as well as datasets from plants subjected to biotic and abiotic stresses. The biotic stress datasets included powdery mildew (PM), angular leaf spot (ALS), cucumber scab (CC), and gray mold (GM), whereas the abiotic stress datasets included heat, cold, and salt treatments. Detailed sample-, experiment-, and run-level metadata are available through the corresponding GSA accession.

Expression values for the 37 *CsTBL* genes were extracted from each dataset, log-transformed, and subsequently standardized within each gene across the corresponding samples before heatmap visualization. Therefore, heatmap colors represent relative changes in the expression pattern of the same gene across samples and should not be interpreted as differences in absolute expression levels among different *CsTBL* genes.

### 2.8. Gene Ontology (GO) Annotation and Enrichment Analysis

To investigate the potential biological functions of the cucumber *CsTBL* gene family, Gene Ontology (GO) annotation and enrichment analyses were performed for the identified *CsTBL* members. First, the CsTBL protein sequences were submitted to the Blast2GO/OmicsBox platform for functional annotation [29]. The sequences were searched against the NCBI non-redundant protein database (NR) using BLASTP, with an E-value threshold of 1 × 10^−5^. Under these criteria, GO terms were successfully assigned to 29 of the 37 CsTBL proteins, whereas eight proteins did not receive GO annotations because no sufficiently informative homologous annotation suitable for GO-term transfer was obtained under the applied criteria.

According to the Gene Ontology classification system, the assigned GO terms were grouped into three major categories: biological process (BP), cellular component (CC), and molecular function (MF) [30]. GO enrichment analysis was therefore performed using the 29 *CsTBL* genes with assigned GO terms as the annotated target set, with annotated protein-coding genes in the cucumber genome used as background references. Statistical significance was assessed using Fisher’s exact test, and multiple-testing correction was performed using the Benjamini–Hochberg procedure. GO terms with an adjusted *p*-value < 0.05 were considered significantly enriched.

For each enriched GO term, the number of associated *CsTBL* genes, enrichment ratio, and statistical significance were calculated. The enrichment results were visualized using TBtools to characterize the biological processes, cellular compartments, and molecular functions potentially associated with the cucumber TBL family.

### 2.9. RT-qPCR Analysis of CsTBL Genes in Cucumber

Cucumber cultivar ‘9930’ seedlings with uniform growth were used as experimental materials. Powdery mildew inoculation was performed when three true leaves were nearly fully expanded. The powdery mildew pathogen *Podosphaera xanthii* (Px) was collected from susceptible cucumber plants and propagated under suitable conditions. For inoculation, fresh conidia were collected from the surfaces of infected leaves and suspended in sterile water to a final concentration of approximately 1 × 10^5^ conidia mL^−1^. The conidial suspension was sprayed evenly onto the cucumber leaf surfaces. After inoculation, the plants were maintained at 25 ± 2 °C and 80–90% relative humidity [31].

After inoculation, the plants were maintained at 25 ± 2 °C and 80–90% relative humidity. At 0 dpi, the second fully expanded true leaves were collected before treatment from separate plant groups designated for subsequent mock and *P. xanthii* treatments. Leaves from mock-treated and *P. xanthii*-inoculated plants were subsequently collected at 1, 3, and 5 dpi. Samples were collected at the same time of day. Three biological replicates were established for each treatment and sampling time point, with each replicate consisting of equal amounts of leaf tissue pooled from the same leaf position of three plants. Samples were immediately frozen in liquid nitrogen and stored at −80 °C until RNA extraction and RT-qPCR analysis.

Based on the descriptive temporal profiles in the public powdery mildew RNA-seq dataset, six *CsTBL* genes (*CsTBL2*, *CsTBL15*, *CsTBL24*, *CsTBL25*, *CsTBL26*, and *CsTBL30*) were selected for independent RT-qPCR analysis. Candidate selection was not based solely on the magnitude of expression change. Instead, genes were chosen to represent contrasting temporal response patterns while considering transcript abundance and complementary evolutionary or functional evidence. Specifically, *CsTBL2* represented sustained repression; *CsTBL15* represented an early increase followed by reduced expression; *CsTBL24* and *CsTBL25* constituted a tandemly duplicated pair with divergent temporal profiles; *CsTBL26* showed progressive middle-to-late accumulation; and *CsTBL30* exhibited a pronounced intermediate-stage peak. *CsTBL15* and *CsTBL26* additionally carried homology-derived annotations associated with xylan O-acetyltransferase activity. Thus, the six genes were selected as representative candidates spanning distinct transcriptional and biological contexts rather than as the six genes with the largest fold changes. Three independent biological replicates were analyzed for each treatment and sampling time point. Total RNA was extracted from cucumber leaves using RNAiso Plus reagent (Takara, Kusatsu, Japan) according to the manufacturer’s instructions. RNA concentration and purity were determined using a NanoDrop spectrophotometer (Thermo Fisher Scientific, Waltham, MA, USA), and RNA integrity was assessed by agarose gel electrophoresis. Only RNA samples showing intact rRNA bands without evident degradation were used for subsequent analysis. Following DNase treatment to remove residual genomic DNA, an equal amount of total RNA (1 μg) from each biological replicate was reverse-transcribed into first-strand cDNA using the PrimeScript™ RT Reagent Kit (Takara, Japan) under identical reaction conditions. The resulting cDNA was diluted tenfold with nuclease-free water and stored at −20 °C until use. Reverse-transcription efficiency was not independently quantified for individual samples; however, equal RNA input and identical reverse-transcription conditions were used for all samples to minimize variation, and the consistency of the resulting cDNA templates was further supported by the stable Ct values of the reference gene *CsActin* across experimental conditions.

Based on previous evaluations of reference-gene stability in cucumber, *CsActin* was selected as the internal reference gene for stress-treated cucumber leaves [32]. Its within-experiment stability was further assessed by examining the raw Ct values across all biological replicates, treatments, and sampling times. No systematic treatment- or time-dependent shift that would compromise its use for normalization was observed. Therefore, CsActin was considered suitable as the reference gene under the experimental conditions used in this study. The corresponding Ct values across experimental conditions are shown in Supplementary Figure S1. Gene-specific RT-qPCR primers were designed using Primer Premier 5.0, and the primer sequences are listed in Table 1. Primer quality was initially evaluated using the Primer Check function in TBtools-II, and amplification specificity was subsequently verified by melting-curve analysis. Standard curves were generated using serially diluted cDNA templates to determine amplification efficiency. Only primer pairs with amplification efficiencies ranging from 90% to 110% were retained. The actual amplification efficiencies of all primer pairs used in this study are provided in Supplementary Table S1.

RT-qPCR was performed using a CFX Connect Real-Time PCR Detection System (Bio-Rad, Hercules, CA, USA) with ArtiCanCEO SYBR qPCR Mix (Tsingke, Beijing, China). Each 20-μL reaction contained 10 μL of ArtiCanCEO SYBR qPCR Mix, 0.4 μL each of forward and reverse primers at 10 μM, 2 μL of diluted cDNA template, and 7.2 μL of ddH_2_O. RT-qPCR was performed using a two-step cycling protocol consisting of an initial denaturation at 95 °C for 5 min, followed by 40 cycles of denaturation at 95 °C for 10 s and combined annealing/extension at 60 °C for 30 s. Melting-curve analysis was performed after amplification using the default instrument settings. Three technical replicates were conducted for each biological replicate, and no-template controls were included to detect reagent contamination, primer-dimer formation, and nonspecific amplification.

Relative expression levels were calculated using the 2^−ΔΔCt^ method [33], where ΔCt was defined as Ct_target − Ct_CsActin and ΔΔCt as ΔCt_sample − mean ΔCt_calibrator. The mean ΔCt of the mock-designated 0 dpi samples was used as a common pre-inoculation calibrator because these samples were collected before treatment, allowing expression values from subsequent mock and *P. xanthii* samples to be reported relative to the same baseline. The 2^−ΔΔCt^ values were used for graphical presentation of relative expression, whereas statistical analyses were performed on ΔCt values at the biological-replicate level. Statistical analyses of the RT-qPCR data were performed using ΔCt values at the biological-replicate level. For each gene, a two-way ANOVA was conducted with treatment (Mock and *P. xanthii*) and time point (0, 1, 3, and 5 dpi) as factors, including the treatment × time interaction. Technical replicates were averaged prior to statistical analysis. The normality of model residuals was assessed using the Shapiro–Wilk test, and no significant departure from normality was detected for any of the six genes (all *p* > 0.05). Tukey-adjusted multiple comparisons were subsequently performed. Full ANOVA statistics, including F values, degrees of freedom, and P values for the main effects and their interaction, are provided in Supplementary Table S2. Statistical analyses and data visualization were conducted using Python v3.14, with Pandas, NumPy, Matplotlib, Seaborn, and statsmodels used for data processing, statistical analysis, and graphical presentation.

## 3. Results

### 3.1. Physicochemical Properties of Cucumber CsTBL Proteins

The physicochemical properties of the 37 cucumber TBL proteins were analyzed. As shown in Supplementary Table S3, the CsTBL proteins ranged from 351 to 624 amino acids in length, with an average length of approximately 444 amino acids. *CsTBL12* was the shortest protein, comprising 351 amino acids, whereas *CsTBL22* was the longest, comprising 624 amino acids. Their predicted molecular weights ranged from 39.72 to 69.81 kDa, with an average of approximately 51.13 kDa. *CsTBL12* had the lowest molecular weight, whereas *CsTBL8* had the highest, indicating variation in protein size among *CsTBL* family members.

The theoretical isoelectric points of the CsTBL proteins ranged from 5.04 to 9.32. *CsTBL26* had the lowest predicted pI, whereas *CsTBL10* had the highest. Among the 37 proteins, 15 had theoretical pI values below 7 and were therefore predicted to be acidic, whereas the remaining 22 had pI values above 7 and were predicted to be basic. These results indicate substantial variation in the predicted charge properties of CsTBL proteins.

The instability indices ranged from 26.39 to 58.51, with *CsTBL11* showing the lowest value and *CsTBL23* the highest. Using an instability index of 40 as the empirical threshold, 14 CsTBL proteins were predicted to be stable, whereas the remaining 23 were predicted to be unstable, accounting for 37.8% and 62.2% of the family, respectively. The aliphatic indices ranged from 59.86 to 89.60. *CsTBL31* had the lowest aliphatic index, whereas *CsTBL12* had the highest, suggesting differences in aliphatic side-chain composition and potentially in thermal stability among family members. The grand average of hydropathicity values was negative for all CsTBL proteins, ranging from −0.688 to −0.109. *CsTBL8* had the lowest GRAVY value, whereas *CsTBL12* had the highest, indicating that the *CsTBL* family is generally hydrophilic.

### 3.2. Phylogenetic Analysis of the Cucumber CsTBL Family

**Figure 1:**
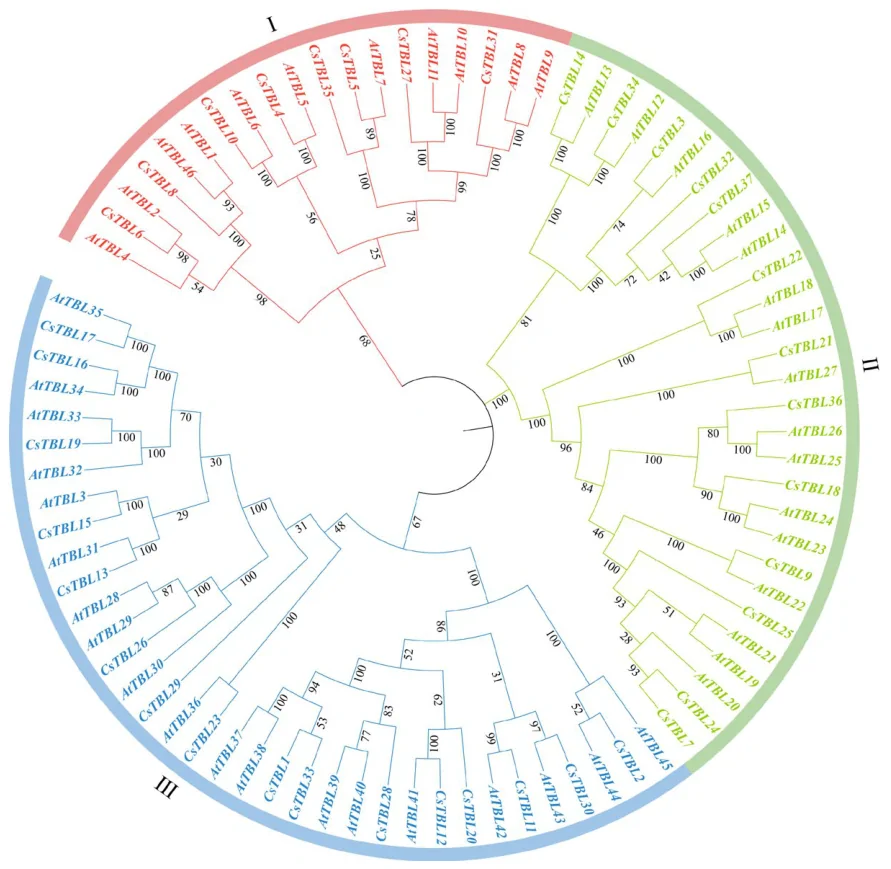
Phylogenetic relationships of TBL proteins from cucumber and *Arabidopsis thaliana*. The phylogenetic tree was constructed in MEGA 11 using the neighbor-joining (NJ) method based on amino acid p-distances from the full-length protein sequences of 37 cucumber CsTBL proteins (*CsTBL1*–*CsTBL37*) and 46 *A. thaliana* AtTBL proteins (*AtTBL1*–*AtTBL46*). Node support was assessed using 1000 bootstrap replicates. Bootstrap values are shown at the corresponding nodes, and Groups I–III are indicated by different colors.

To investigate the evolutionary relationships between the cucumber and *Arabidopsis* TBL families, a phylogenetic tree was constructed using 46 AtTBL and 37 CsTBL protein sequences. As shown in Figure 1, based on the topology of the NJ tree, the 83 TBL proteins were descriptively divided into three major groups, designated Groups I, II, and III. Bootstrap support varied among internal nodes; therefore, relationships supported by bootstrap values ≥70% were given greater weight in interpreting the phylogenetic associations. These groups contained 19, 29, and 35 members, respectively. Of the 37 CsTBL proteins, 8, 13, and 16 were assigned to Groups I, II, and III, respectively. Each group contained both cucumber and Arabidopsis proteins, and members from the two species were interspersed throughout the tree rather than forming distinct species-specific clusters. This pattern suggests that substantial diversification of the TBL family occurred before the divergence of cucumber and *Arabidopsis* and that multiple TBL lineages have remained evolutionarily conserved in these two dicot species.

Several CsTBL proteins formed cross-species sister-group relationships with AtTBL proteins. In Group I, *CsTBL6*, *CsTBL4*, and *CsTBL10* clustered with *AtTBL2*, *AtTBL5*, and *AtTBL6*, respectively. In Group II, *CsTBL34*, *CsTBL14*, *CsTBL21*, and *CsTBL9* formed sister groups with *AtTBL12*, *AtTBL13*, *AtTBL27,* and *AtTBL22*, respectively. In Group III, *CsTBL16*, *CsTBL17*, *CsTBL19*, *CsTBL15*, and *CsTBL13* clustered with *AtTBL34*, *AtTBL35*, *AtTBL33*, *AtTBL3*, and *AtTBL31*, respectively. *CsTBL23*, *CsTBL12*, *CsTBL11*, and *CsTBL30* also showed close phylogenetic relationships with *AtTBL36*, *AtTBL41*, *AtTBL42*, and *AtTBL43*, respectively. These cross-species relationships indicate a relatively high degree of sequence conservation between the corresponding cucumber and Arabidopsis proteins and suggest that some of these CsTBL proteins may have retained functions similar to those of their *AtTBL* counterparts.

Several cucumber proteins also formed intraspecific sister groups, including *CsTBL1* with *CsTBL33* and *CsTBL7* with *CsTBL24*, whereas some subclades contained multiple *CsTBL* members. These patterns suggest that the cucumber TBL family may have undergone lineage-specific gene duplication and duplicate retention, potentially followed by functional divergence. However, this interpretation requires further support from chromosomal localization, duplication-mode classification, and synteny analyses. Overall, the phylogenetic analysis revealed evolutionary diversification within the cucumber TBL family and identified potential homologous relationships between CsTBL and AtTBL proteins, thereby providing a basis for subsequent functional prediction and candidate-gene selection.

### 3.3. Conserved Motif and Gene Structure Analyses of the Cucumber CsTBL Family

**Figure 2:**
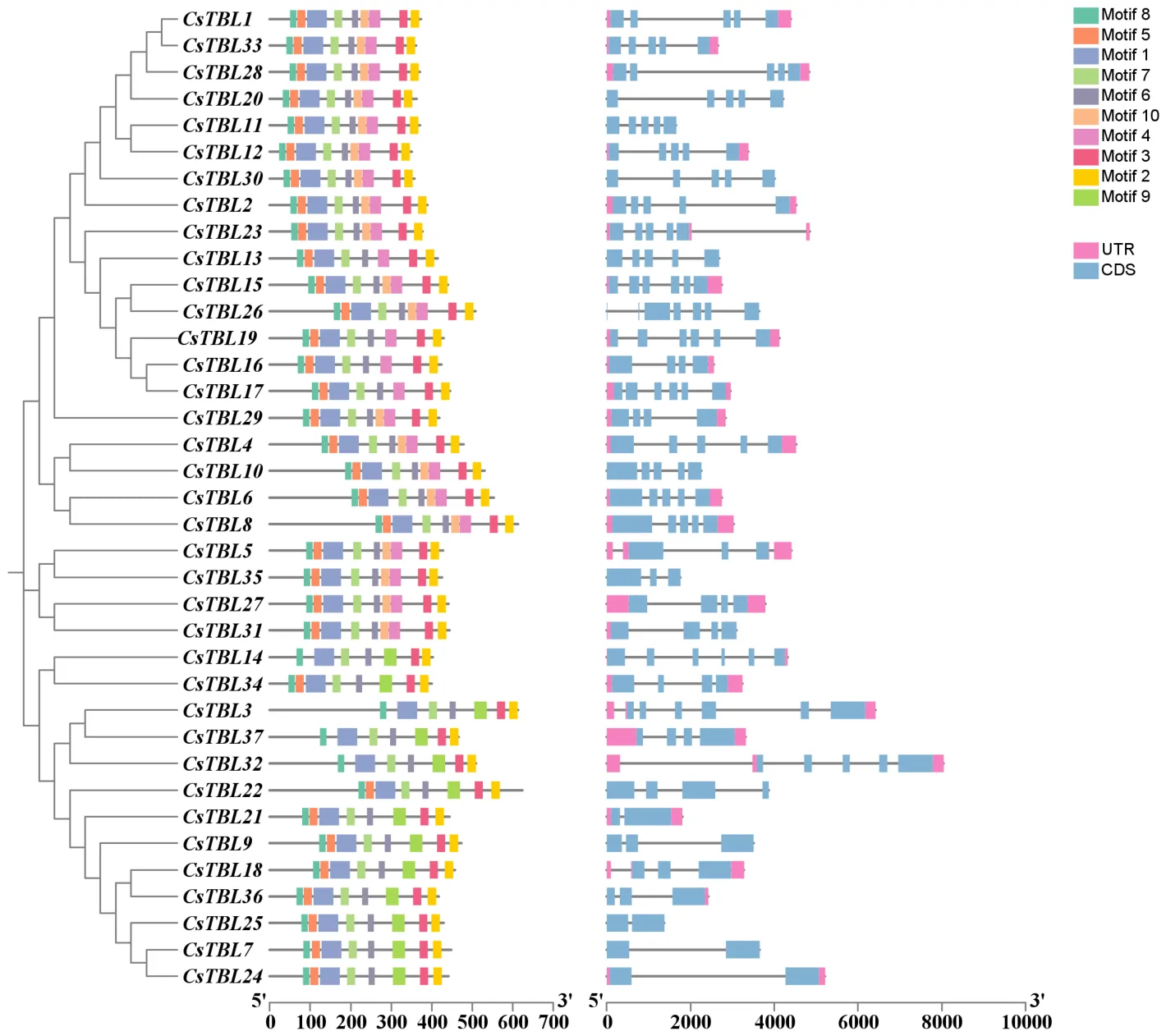
Phylogenetic relationships, conserved motif composition, and gene structures of cucumber TBL family members. The left panel shows the phylogenetic tree constructed from the protein sequences of 37 *CsTBL* family members. The middle panel shows the composition and distribution of ten conserved motifs (Motifs 1–10) in CsTBL proteins. Rectangles of different colors represent different motifs, and their positions indicate the relative locations of the corresponding motifs within each protein sequence. The horizontal axis represents protein length in amino acids (aa). The right panel shows the exon–intron structures of the *CsTBL* genes. Pink rectangles represent untranslated regions (UTRs), blue rectangles represent coding sequences (CDSs), and connecting lines represent introns. The horizontal axis indicates genomic sequence length in base pairs (bp). Gene models are displayed in the 5′-to-3′ direction, whereas protein sequences are displayed from the N- to the C-terminus.

Conserved motifs in the 37 CsTBL proteins were analyzed using MEME. As shown in Figure 2, ten conserved motifs, designated Motifs 1–10, were identified, with lengths ranging from 16 to 50 amino acid residues. The occurrence frequencies of these motifs varied considerably among CsTBL proteins. Motifs 1, 2, 3, 6, 7, and 8 were present in all 37 CsTBL proteins, indicating that they are highly conserved and may constitute the common core sequence features of the family. Motif 5 was detected in 33 members and was also highly conserved. By contrast, Motif 4 was present in only 24 members, whereas Motifs 10 and 9 were detected in 16 and 12 members, respectively, indicating more restricted distributions.

The conserved motifs also differed in length and statistical significance. Motif 1 was the longest, comprising 50 amino acid residues, and showed the highest information content and strongest statistical support. Motif 6 was the shortest, comprising 16 amino acid residues, whereas the remaining motifs ranged from 17 to 32 amino acids in length. All motifs had very low E-values, indicating strong statistical support under the current dataset and parameter settings. Motifs 1, 2, and 3 were detected in all CsTBL proteins and showed particularly strong statistical support, further demonstrating the high degree of sequence conservation within the *CsTBL* family.

In the gene structure diagram, untranslated regions (UTRs) are shown in pink, coding sequences (CDSs) in blue, and connecting lines indicate the intervening genomic regions. All 37 *CsTBL* genes contained CDS regions, although the number and length of CDS segments varied among members. UTRs were displayed at the 5′ end, 3′ end, or both ends of some genes, whereas no UTRs were shown for other genes. The lengths and positions of the annotated UTRs also varied among family members.

**Figure 3:**
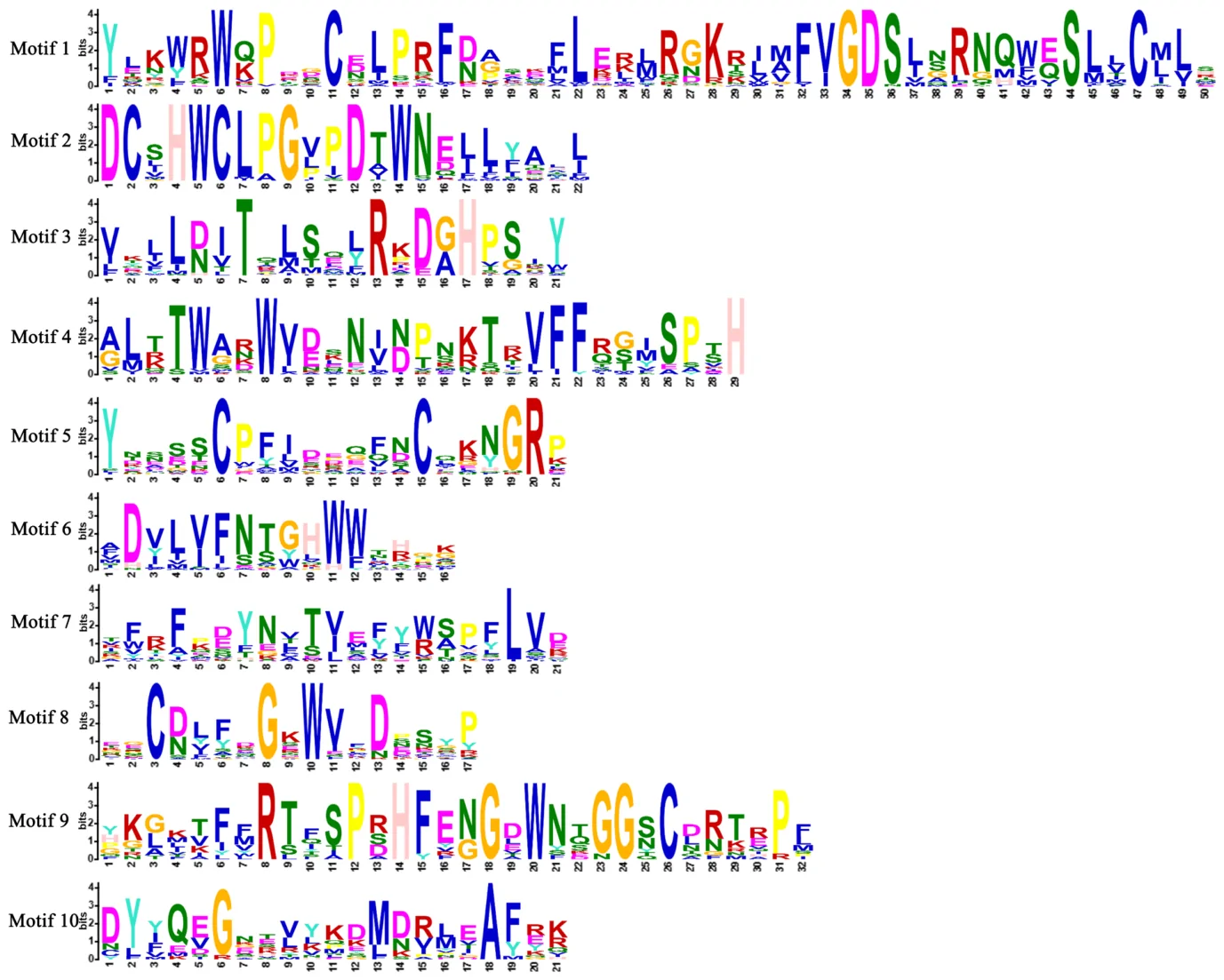
Sequence logos of conserved motifs in cucumber TBL proteins. Ten conserved motifs (Motifs 1–10) were identified from the amino acid sequences of 37 CsTBL proteins using MEME, and their sequence logos were generated. The horizontal axis indicates amino acid positions within each motif, and the vertical axis represents information content in bits. The total height of the letters at each position reflects the degree of sequence conservation, whereas the relative height of each individual letter indicates the frequency of the corresponding amino acid at that position. Different colors denote different classes of amino acids.

Sequence-logo analysis in Figure 3 reveals strong conservation of the characteristic GDS- and DxxH-containing regions across the *CsTBL* family. Motif 1 contained a highly conserved GDS sequence, whereas the N-terminal region of Motif 2 contained a prominent DCSH sequence corresponding to the characteristic DxxH motif of TBL proteins. Motifs 1 and 2 were detected in all 37 CsTBL proteins, supporting conservation of the characteristic TBL core motif architecture. In this context, DCSH represents a specific sequence realization of the broader DxxH motif, in which the defining Asp and His residues retain their characteristic spacing. However, the functional significance of the intervening residues cannot be determined from sequence conservation alone. The full-length multiple sequence alignment of all 37 CsTBL proteins is provided in Supplementary Data S1 in aligned FASTA format to facilitate examination of sequence conservation and lineage-specific variation among individual family members. Motifs 3, 6, 7, and 8 were also detected in all CsTBL proteins and contained multiple highly conserved residues, further supporting the presence of a stable core motif framework across the family.

By contrast, Motifs 4, 9, and 10 showed pronounced member- or clade-specific distributions. Motif 10 was detected only in a subset of proteins containing Motif 4, whereas Motif 9 showed a mutually exclusive distribution with Motifs 4 and 10, suggesting that distinct combinations of variable motifs have evolved among different *CsTBL* lineages. Motif 5 was present in most family members but was not detected in *CsTBL3*, *CsTBL14*, *CsTBL32*, or *CsTBL37*. Collectively, these results indicate that CsTBL proteins retain the conserved GDS and DxxH motifs and a shared core motif architecture, while some members have diverged in their accessory motif composition. Such sequence variation may provide a structural basis for functional diversification within the family.

### 3.4. Structural Characterization of CsTBL Proteins

Computational secondary-structure prediction indicated that random coils were the predominant predicted structural element in the 37 CsTBL proteins (49.72–71.62%), followed by α-helices and extended strands, whereas β-turns were least abundant. Homology models generated by SWISS-MODEL suggested broadly similar folded core regions among several family members but also variation in predicted peripheral regions. These structural features should be interpreted cautiously because they are computational predictions rather than experimentally resolved structures. In particular, membrane-associated topology, flexible regions, and poorly conserved peripheral segments may not be reliably represented by template-based homology modeling. Detailed secondary- and tertiary-structure predictions are provided in Supplementary Figures S2 and S3 and Table S4.

### 3.5. Analysis of Cis-Regulatory Elements in the Promoters of Cucumber CsTBL Genes

**Figure 4:**
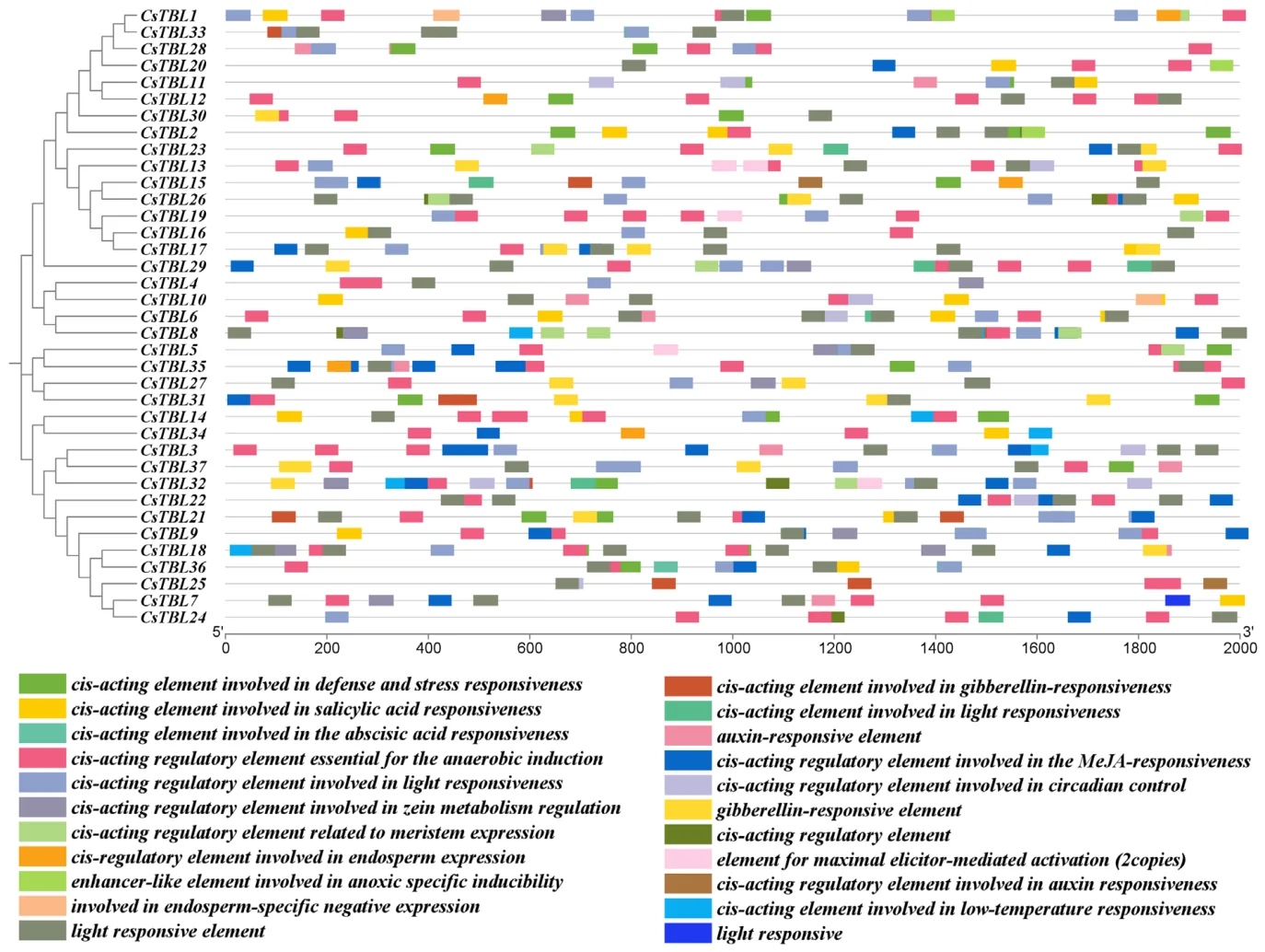
Distribution of PlantCARE-predicted cis-regulatory elements in the promoters of cucumber *CsTBL* genes. The 2000-bp sequences upstream of the start codons of the 37 *CsTBL* genes were analyzed using PlantCARE. The dendrogram on the left shows clustering of *CsTBL* promoters based on their predicted cis-element profiles. Colored rectangles indicate the positions of predicted cis-regulatory elements along each promoter, with each color corresponding to the original functional annotation provided by PlantCARE and listed in the legend. The horizontal axis indicates position within the 2000-bp promoter sequence, displayed in the 5′-to-3′ direction. PlantCARE annotation labels were retained according to the original database output; therefore, closely related annotations may appear as separate entries in the legend. These elements represent computationally predicted regulatory motifs and do not by themselves demonstrate experimentally validated regulatory activity.

PlantCARE analysis identified 556 predicted cis-regulatory element occurrences within the 2000-bp upstream regions of the 37 *CsTBL* genes (Figure 4). The identified elements represented 22 PlantCARE annotation labels, with frequently detected annotations related to anaerobic induction, light responsiveness, methyl jasmonate (MeJA) responsiveness, abscisic acid responsiveness, and defense and stress responsiveness. Additional predicted elements were annotated in relation to salicylic acid, gibberellin and auxin responses, low-temperature responsiveness, anoxic inducibility, meristem expression, endosperm expression, circadian control, and elicitor-mediated activation. The complete set of PlantCARE annotation labels is shown in the legend of Figure 4.

The abundance and spatial distribution of predicted cis-regulatory elements differed among *CsTBL* promoters, indicating heterogeneous promoter architectures across the family. These patterns provide candidate regulatory features that may be relevant to the diverse expression profiles observed among *CsTBL* genes. However, PlantCARE annotations represent computationally predicted cis-regulatory motifs rather than experimentally validated regulatory interactions. Therefore, the presence of a particular element should not be interpreted as direct evidence that the corresponding *CsTBL* gene responds to a specific hormone, stress, or developmental signal.

### 3.6. Chromosomal Distribution of the Cucumber CsTBL Gene Family

**Figure 5:**
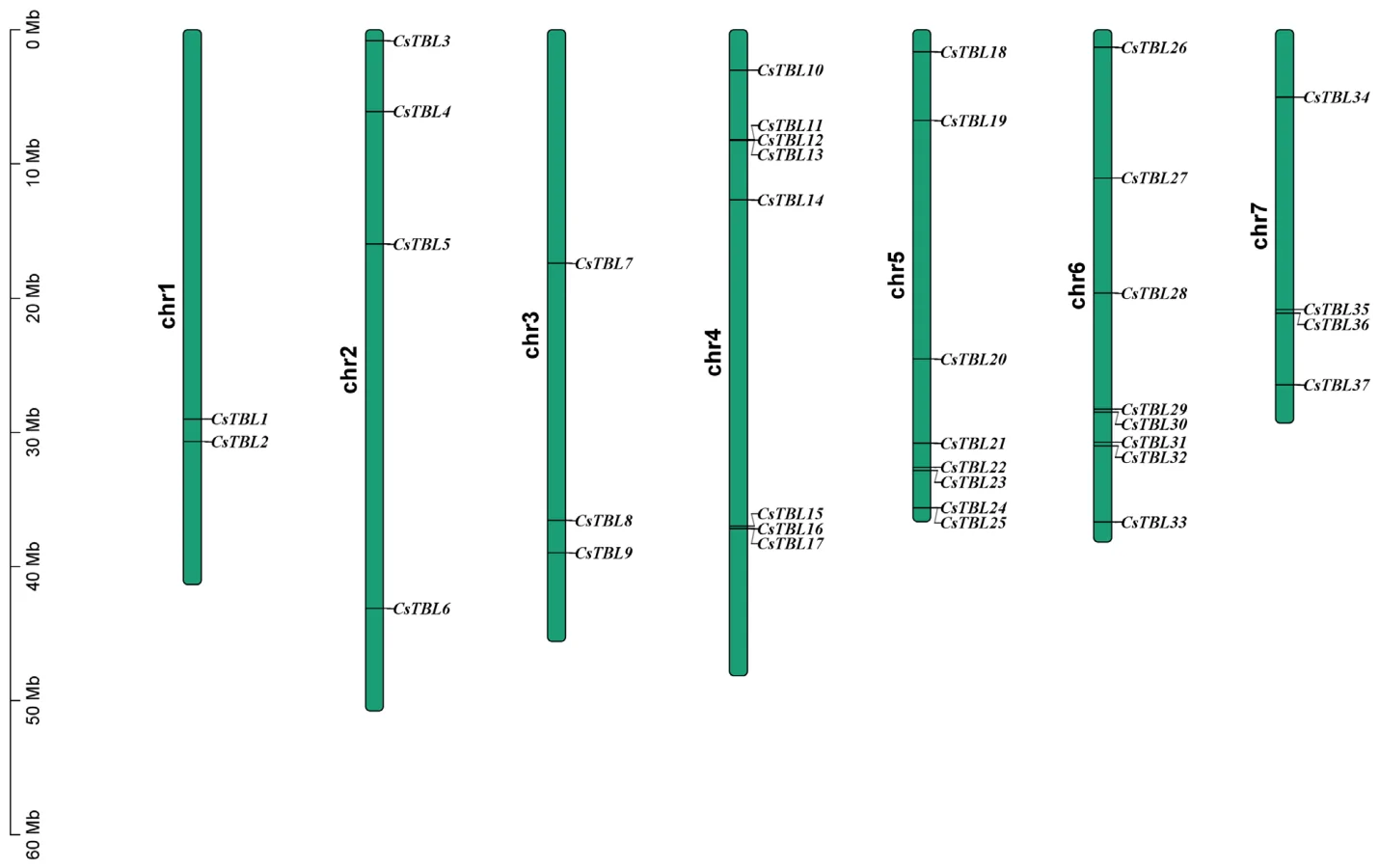
Chromosomal distribution of cucumber *CsTBL* gene family members. The 37 *CsTBL* genes were mapped onto the seven cucumber chromosomes. Green bars represent chromosomes, and gene names with corresponding short lines indicate the physical positions of the *CsTBL* genes. The scale on the left indicates chromosome length in megabases (Mb).

Based on the cucumber genome annotation, the chromosomal locations of the 37 *CsTBL* genes were determined. As shown in Figure 5, all *CsTBL* genes were mapped to the seven cucumber chromosomes, but their distribution was uneven. Chromosomes 4, 5, and 6 contained the largest numbers of *CsTBL* genes, with eight members on each chromosome. Chromosomes 2 and 7 each contained four members, chromosome 3 contained three, and chromosome 1 contained only two.

The physical positions of the *CsTBL* genes also varied considerably among chromosomes. On chromosome 1, *CsTBL1* and *CsTBL2* were located close to each other in the lower region. On chromosome 2, *CsTBL3*, *CsTBL4*, *CsTBL5*, and *CsTBL6* were distributed across different regions and were relatively dispersed. On chromosome 3, *CsTBL7* was located in the middle region, whereas *CsTBL8* and *CsTBL9* were clustered in the lower region. On chromosome 4, *CsTBL10–CsTBL14* were mainly distributed in the upper region, whereas *CsTBL15–CsTBL17* were concentrated in the lower region. On chromosome 5, *CsTBL18* and *CsTBL19* were located in the upper region, *CsTBL20* in the middle region, and *CsTBL21–CsTBL25* in the lower region. On chromosome 6, *CsTBL26–CsTBL28* were distributed from the upper to the middle region, whereas *CsTBL29–CsTBL33* were mainly located in the lower region. On chromosome 7, *CsTBL34* was located in the upper region, *CsTBL35* and *CsTBL36* were positioned close to each other, and *CsTBL37* was located in the lower region.

Overall, the *CsTBL* genes showed an uneven distribution across the seven cucumber chromosomes. Some members were clustered within neighboring chromosomal regions, whereas others were more widely dispersed.

### 3.7. Collinearity Analysis of the Cucumber CsTBL Gene Family

**Figure 6:**
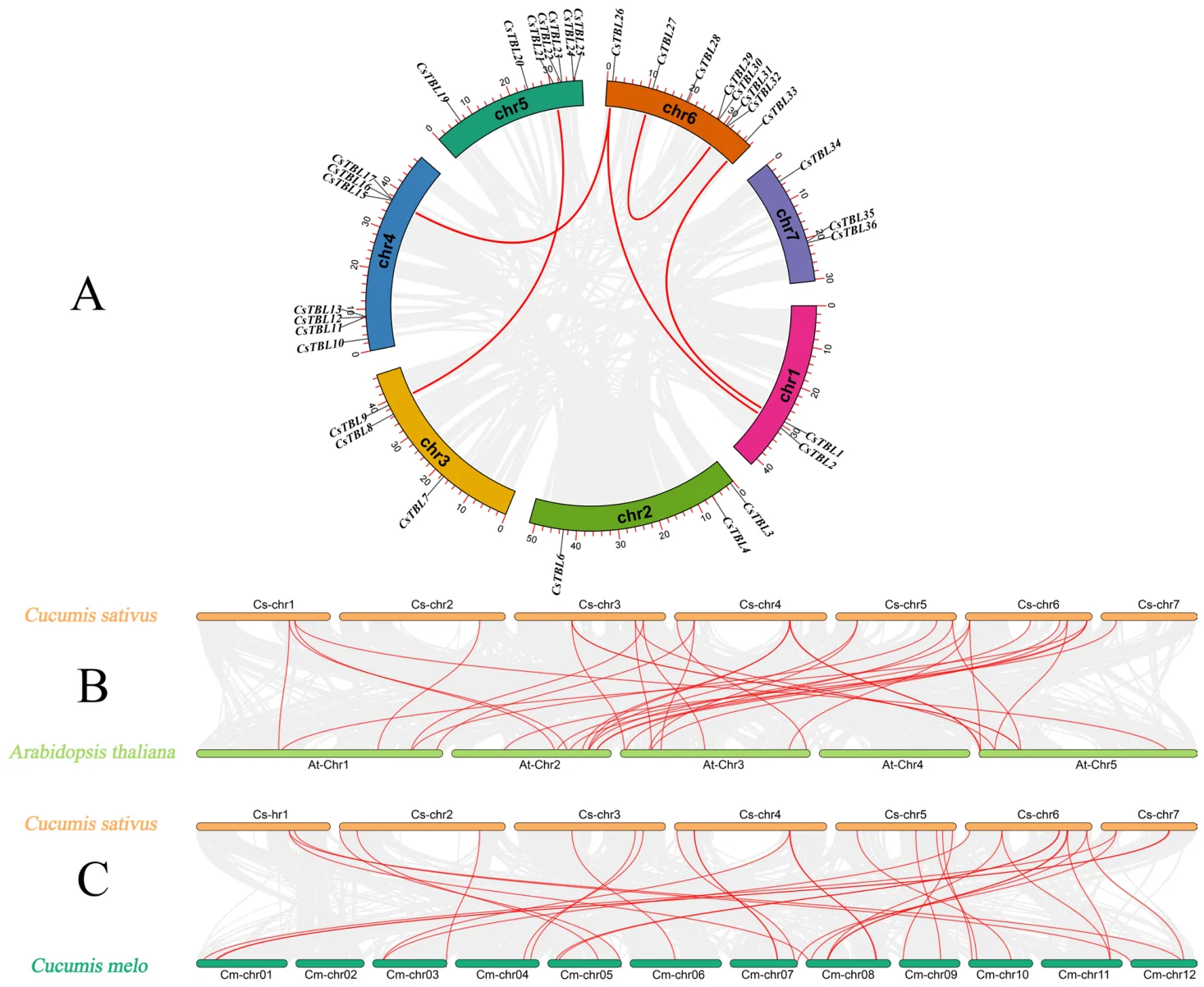
Intraspecific and interspecific syntenic relationships of the cucumber *CsTBL* gene family. (**A**) Intraspecific synteny analysis of *CsTBL* family members within the cucumber genome. Circular segments in different colors represent the seven cucumber chromosomes, and the outer scale indicates chromosomal positions in megabases (Mb). Gray lines represent syntenic blocks within the cucumber genome, whereas red lines indicate syntenic relationships among *CsTBL* family members. (**B**) Interspecific syntenic relationships between TBL family members in cucumber (*Cucumis sativus*) and Arabidopsis thaliana. Orange and green bars represent the chromosomes of cucumber and Arabidopsis, respectively. Gray lines indicate syntenic blocks between the two genomes, and red lines indicate syntenic relationships between *CsTBL* and *AtTBL* genes. (**C**) Interspecific syntenic relationships between TBL family members in cucumber and melon (*Cucumis melo*). Orange and green bars represent the chromosomes of cucumber and melon, respectively. Gray lines indicate syntenic blocks between the two genomes, and red lines indicate syntenic relationships between *CsTBL* genes and their corresponding *TBL* genes in melon.

To investigate the duplication patterns of *CsTBL* family members and their conservation across species, collinearity analyses were performed within the cucumber genome and between cucumber and *Arabidopsis* or melon. As shown in Figure 6, multiple collinear relationships were detected among *CsTBL* genes within the cucumber genome, including *CsTBL1–CsTBL33*, *CsTBL2–CsTBL26*, *CsTBL9–CsTBL21*, *CsTBL15–CsTBL26*, and *CsTBL27–CsTBL31*. Several duplication modes, including dispersed, whole-genome/segmental, and tandem duplication, were identified within the *CsTBL* family. Tandemly duplicated gene pairs included *CsTBL11–CsTBL12*, *CsTBL16–CsTBL17*, and *CsTBL24–CsTBL25*. These results indicate that multiple duplication mechanisms contributed to the expansion of the *CsTBL* family in the cucumber genome.

Interspecific collinearity analyses further showed that *CsTBL* genes retained syntenic relationships with *TBL* genes in both *Arabidopsis* and melon. Multiple *CsTBL*–*AtTBL* syntenic gene pairs were identified between cucumber and Arabidopsis, whereas more extensive syntenic relationships were observed between cucumber and melon. One-to-many and many-to-many relationships were detected in both interspecific comparisons, consistent with differential gene duplication and retention during species divergence. However, these syntenic relationships should not be interpreted as definitive one-to-one orthology. Rather, they identify candidate orthologous or co-retained genomic relationships that may also include duplicated paralogous copies. Collectively, these results indicate that the *CsTBL* family underwent duplication-driven expansion within the cucumber genome and retained varying degrees of synteny with *TBL* genes in Arabidopsis and melon. These patterns are consistent with both conservation of ancestral genomic relationships and lineage-specific duplication, retention, loss, and rearrangement. However, the observed interspecific syntenic links should not be interpreted as definitive evidence of one-to-one orthology, particularly for one-to-many and many-to-many relationships.

### 3.8. Expression Patterns of CsTBL Genes

#### 3.8.1. Tissue- and Developmental-Stage-Dependent Expression Profiles

**Figure 7:**
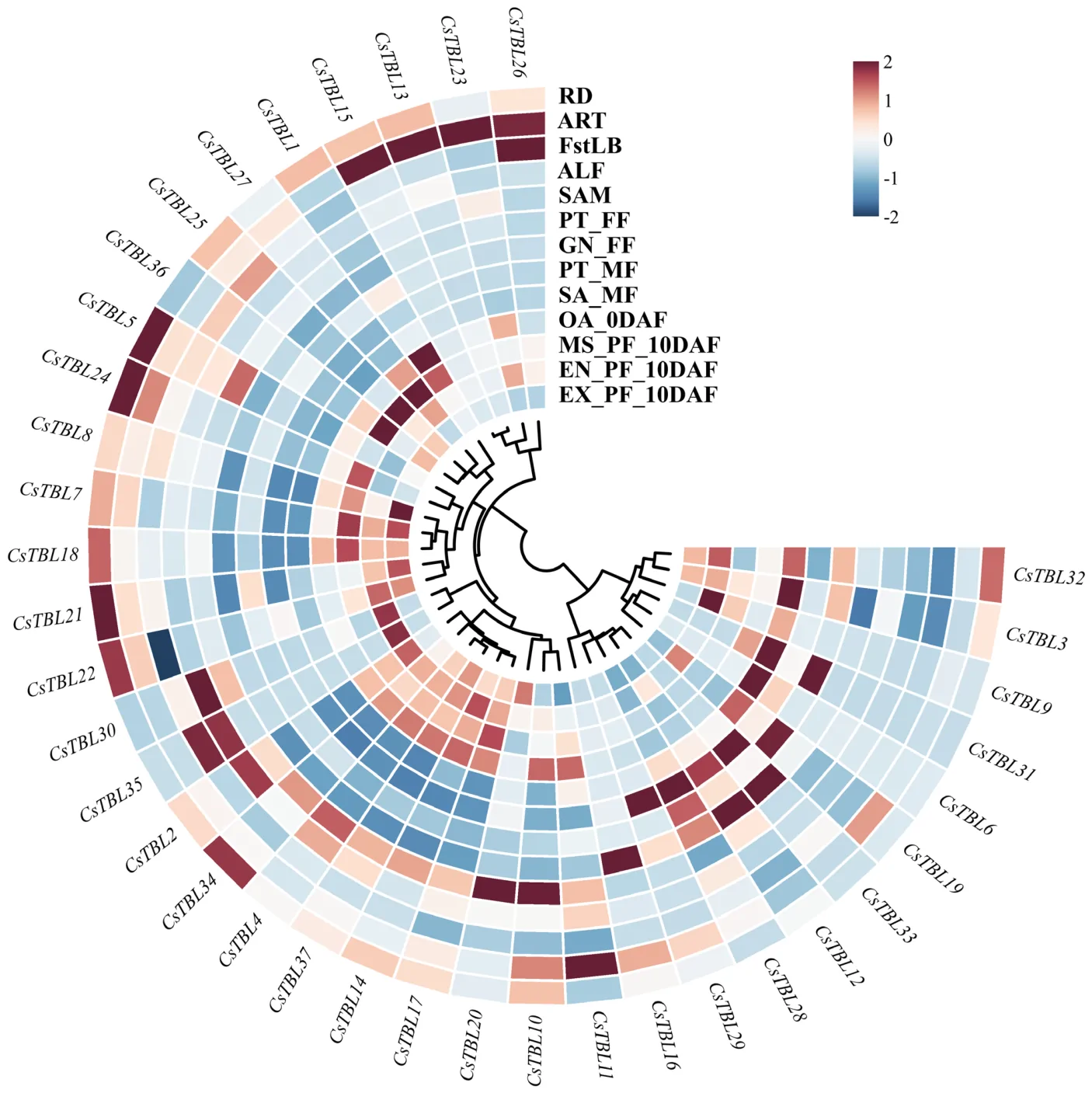
Expression patterns of the *CsTBL* gene family across different tissues and developmental stages. Based on cucumber transcriptomic data, the expression levels of 37 *CsTBL* genes in roots, leaves, shoot apical meristems, female and male floral organs, ovaries at anthesis, and different fruit tissues after pollination were subjected to clustering analysis. In the circular heatmap, the outer ring shows the names of the *CsTBL* genes, and the radial tracks represent samples from different tissues or developmental stages. The dendrogram in the center indicates hierarchical clustering relationships based on gene expression patterns. Colors represent gene-wise relative expression levels, with red indicating relatively high expression and blue indicating relatively low expression. Abbreviations for tissue and developmental-stage labels are defined in abbreviations at the end of the text.

The expression patterns of *CsTBL* family members across different tissues and developmental stages were analyzed by hierarchical clustering. As shown in Figure 7, the genes exhibited markedly different expression profiles and formed several tissue-preferential expression modules. *CsTBL13*, *CsTBL15*, *CsTBL23*, and *CsTBL26* showed relatively high expression in root- or leaf-related samples. *CsTBL6*, *CsTBL12*, *CsTBL16*, *CsTBL19*, *CsTBL28*, *CsTBL29*, *CsTBL31*, and *CsTBL33* were expressed predominantly in female or male floral organs, with individual genes showing distinct expression preferences among the corolla, gynoecium, and anther.

*CsTBL1*, *CsTBL7*, *CsTBL8*, *CsTBL17*, *CsTBL18*, *CsTBL21*, *CsTBL24*, *CsTBL25*, *CsTBL27*, and *CsTBL36* exhibited relatively high expression in ovary- or fruit-related tissues. Several members displayed distinct expression patterns among the mesocarp, endocarp, and exocarp, indicating pronounced spatial variation in their relative transcript profiles within the fruit. The expression levels of *CsTBL3*, *CsTBL22*, and *CsTBL32* varied relatively little across the examined samples, whereas some other genes showed high expression in only a limited number of tissues.

Overall, *CsTBL* family members exhibited distinct expression patterns across vegetative organs, floral organs, ovaries, and fruit tissues, indicating marked spatiotemporal differentiation in gene expression. The colors in the heatmap represent gene-wise standardized relative expression levels and are intended only for comparing the expression pattern of each gene across different samples.

#### 3.8.2. Expression Profiles of CsTBL Genes Under Biotic Stress

**Figure 8:**
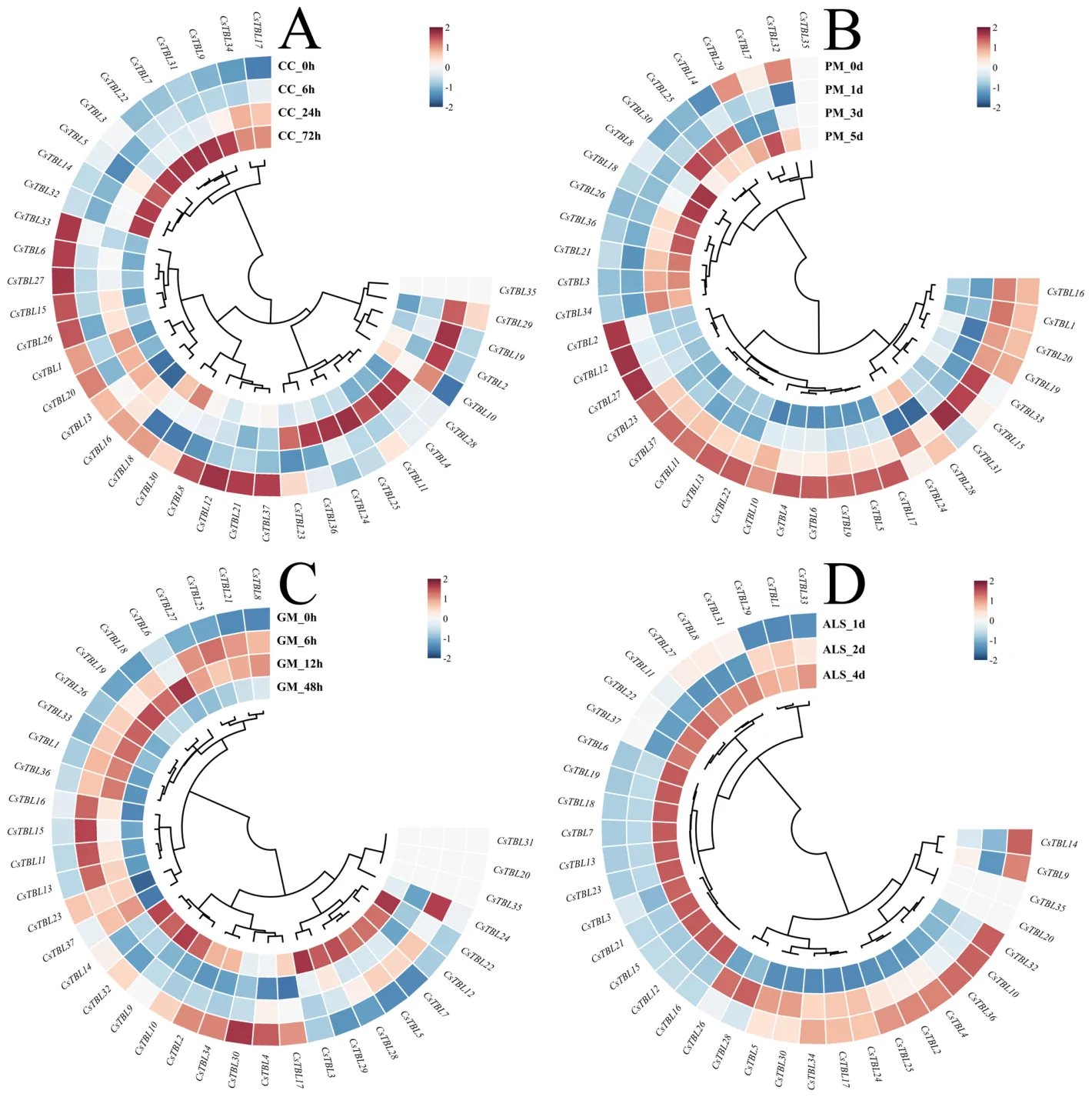
Dynamic expression patterns of the *CsTBL* gene family under different disease treatments. (**A**) Cucumber scab (CC) at 0, 6, 24, and 72 h after treatment; (**B**) powdery mildew (PM) at 0, 1, 3, and 5 d after treatment; (**C**) gray mold (GM) at 0, 6, 12, and 48 h after treatment; and (**D**) angular leaf spot (ALS) at 1, 2, and 4 d after treatment. In each panel, the concentric rings from the outside inward represent the corresponding sampling time points. *CsTBL* gene names are shown around the outer edge, and the central dendrogram indicates the hierarchical clustering relationships based on expression patterns under each treatment condition. Colors represent relative gene expression levels, with red indicating relatively high expression and blue indicating relatively low expression. The clustering branches in different panels are independent of one another, and the colors are intended only to compare the relative expression changes of the same gene across the time course within each treatment.

As shown in Figure 8, the *CsTBL* family exhibited pronounced time-dependent expression changes in response to four pathogen treatments. A conventional rectangular heatmap is provided in Supplementary Figure S4. The *CsTBL* family exhibited pronounced time-dependent expression changes in response to four pathogen treatments, although the response patterns differed among diseases. During cucumber scab infection, most responsive genes remained at relatively low expression levels during the early stages and increased at later time points, indicating a delayed response. This pattern was observed for *CsTBL4*, *CsTBL7*, *CsTBL9*, *CsTBL10*, *CsTBL14*, *CsTBL22*, and *CsTBL34*. *CsTBL6*, *CsTBL15*, *CsTBL26*, *CsTBL27*, and *CsTBL33* showed relatively high expression at the initial stage, followed by a gradual decline.

Within the powdery-mildew-inoculated time series, *CsTBL* genes displayed several relative temporal expression patterns. One group, including *CsTBL1*, *CsTBL16*, *CsTBL19*, *CsTBL26*, and *CsTBL29*, showed relatively lower standardized expression at 1 d followed by higher values at 3 or 5 d. A second group, including *CsTBL2*, *CsTBL12*, and *CsTBL27*, showed relatively high standardized expression at 0 d and lower values at subsequent sampling times. During gray mold infection, many genes showed increased relative expression at 6–12 h after treatment but decreased expression at 48 h, indicating a transient early response. Other members exhibited relatively high expression only at 48 h, suggesting the presence of both early- and late-responsive expression modules. Under angular leaf spot infection, most *CsTBL* genes shifted from relatively low expression at 1 day after treatment to relatively high expression at 2–4 days, displaying a broadly consistent late-response pattern.

Overall, the *CsTBL* family exhibited distinct dynamic expression patterns in response to different diseases. Late-stage responses were particularly prominent under cucumber scab and angular leaf spot treatments, whereas powdery mildew treatment induced both recovery-associated increases and sustained decreases in expression. Gray mold treatment was characterized mainly by transient early responses. These findings indicate substantial variation in the transcriptional responses of individual *CsTBL* genes among pathogen types and infection stages.

#### 3.8.3. Expression Profiles of CsTBL Genes Under Abiotic Stress

**Figure 9:**
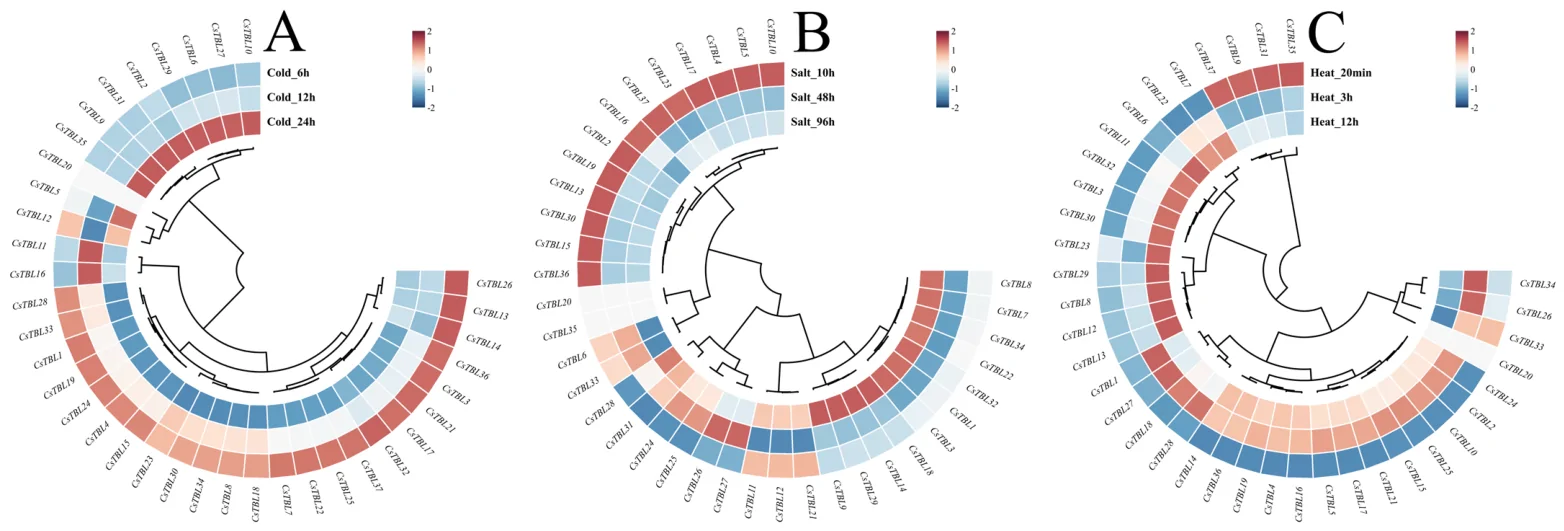
Dynamic expression patterns of the *CsTBL* gene family under cold, salt, and heat stress. (**A**) Cold stress at 6, 12, and 24 h after treatment; (**B**) salt stress at 10, 48, and 96 h after treatment; and (**C**) heat stress at 20 min, 3 h, and 12 h after treatment. *CsTBL* gene names are shown around the outer edge of the circular heatmap, and different concentric rings represent different time points under each stress treatment. The central dendrogram indicates hierarchical clustering relationships based on expression patterns. Red and blue indicate relatively high and relatively low expression levels, respectively, of the same gene across different treatment time points.

The expression patterns of the 37 *CsTBL* genes under cold, salt, and heat stress were analyzed. As shown in Figure 9, the *CsTBL* family exhibited pronounced time-dependent responses to all three stresses. A conventional rectangular heatmap is provided in Supplementary Figure S5. The *CsTBL* family exhibited pronounced time-dependent responses to all three stresses, although the predominant expression patterns differed among treatments. Under cold stress, several genes showed delayed responses. For example, *CsTBL27* gradually increased in expression with prolonged treatment, whereas *CsTBL1* and *CsTBL24* showed sustained decreases. Under salt stress, both early and progressive response patterns were observed. *CsTBL2* and *CsTBL10* displayed relatively high expression during the early stage followed by a decline, whereas *CsTBL28* gradually increased in expression over time. Under heat stress, most responsive genes could be classified into two major patterns: transient induction at the intermediate stage and late-stage accumulation. *CsTBL26* and *CsTBL34* were mainly induced at 3 h, whereas *CsTBL3* and *CsTBL30* reached relatively high expression levels at 12 h.

Overall, individual *CsTBL* genes exhibited diverse dynamic patterns under cold, salt, and heat stress, including early responses, delayed responses, sustained induction, and sustained repression. The direction of expression change for the same gene also varied among stress treatments, indicating marked stress- and time-specific transcriptional responses within the *CsTBL* family.

### 3.9. Gene Ontology Enrichment Analysis

**Figure 10:**
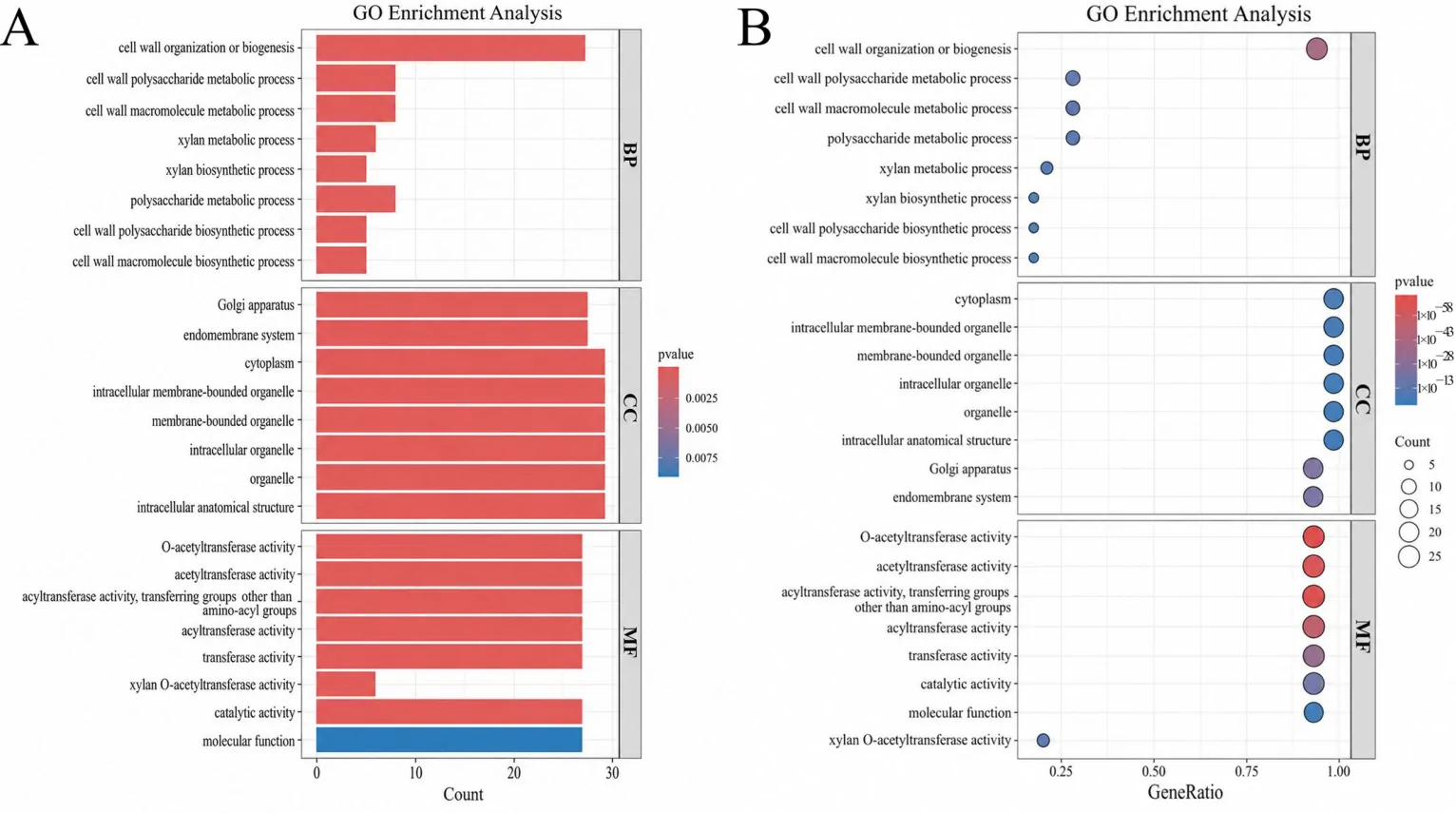
Gene Ontology enrichment analysis of the cucumber *CsTBL* gene family. (**A**) GO enrichment analysis of *CsTBL* genes with assigned GO annotations, classified into biological process (BP), cellular component (CC), and molecular function (MF) categories. The vertical axis shows significantly enriched GO terms, and the horizontal axis indicates the number of genes associated with each term (Count). Bar color represents the significance of enrichment (*p*-value). The *CsTBL* genes were primarily enriched in terms related to cell-wall organization or biogenesis, cell-wall polysaccharide and xylan metabolism, the Golgi apparatus and endomembrane system, and O-acetyltransferase and acyltransferase activities. (**B**) Representative enriched GO terms in the BP, CC, and MF categories. The vertical axis shows the GO terms, and the horizontal axis indicates the proportion of genes associated with each term (GeneRatio). Bubble size represents the number of genes associated with each term (Count), and bubble color indicates enrichment significance (*p*-value). The *CsTBL* genes showed pronounced enrichment in functional categories associated with cell-wall formation and polysaccharide metabolism, localization to the Golgi apparatus and endomembrane system, and O-acetyltransferase and xylan O-acetyltransferase activities.

To further predict the potential biological functions of the *CsTBL* family, Gene Ontology (GO) enrichment analysis was performed on the 29 CsTBL proteins with assigned GO annotations. The remaining eight CsTBL proteins lacked transferable GO annotations under the applied Blast2GO criteria and were therefore not represented in the enrichment analysis. Consequently, the enrichment results describe the functional tendencies of the annotated subset rather than providing complete functional coverage of all 37 *CsTBL* family members. As shown in Figure 10, *CsTBL* genes were mainly enriched in functional categories associated with cell-wall organization and biogenesis, polysaccharide metabolism, the Golgi apparatus, and acetyltransferase activity.

Within the biological process category, *CsTBL* genes were significantly enriched in cell-wall organization or biogenesis, which included 27 genes, accounting for 93.1% of the annotated family members, and showed a high enrichment level. Other significantly enriched terms included cell-wall polysaccharide metabolic process, xylan metabolic process, and xylan biosynthetic process, suggesting that the *CsTBL* family may participate in the biosynthesis and modification of cell-wall polysaccharides.

Within the cellular component category, Golgi apparatus and endomembrane system were significantly enriched, each containing 27 genes, indicating that most CsTBL proteins may be associated with the Golgi apparatus or other components of the endomembrane system. Within the molecular function category, O-acetyltransferase activity was the most significantly enriched term and included 27 genes. A subset of proteins was also associated with xylan O-acetyltransferase activity, including *CsTBL13*, *CsTBL15*, *CsTBL16*, *CsTBL17*, *CsTBL19*, and *CsTBL26*.

Collectively, the GO enrichment results suggest that the *CsTBL* family is primarily involved in the O-acetylation of cell-wall polysaccharides within the Golgi-associated endomembrane system. In particular, *CsTBL13*, *CsTBL15*, *CsTBL16*, *CsTBL17*, *CsTBL19*, and *CsTBL26* represent putative xylan O-acetyltransferases.

### 3.10. CsTBL Genes Exhibit Distinct Expression Patterns in Response to Podosphaera xanthii Infection

To independently examine genes representing contrasting temporal profiles in the descriptive public powdery mildew dataset, six candidate *CsTBL* genes—*CsTBL2*, *CsTBL15*, *CsTBL24*, *CsTBL25*, *CsTBL26*, and *CsTBL30*—were analyzed by RT-qPCR. Transcript abundance was measured at 0, 1, 3, and 5 days post-inoculation (dpi) following *Podosphaera xanthii* inoculation. Relative expression levels were calculated using the 2^−ΔΔCt^ method, with the mean pre-inoculation expression level at 0 dpi as the calibrator.

**Figure 11:**
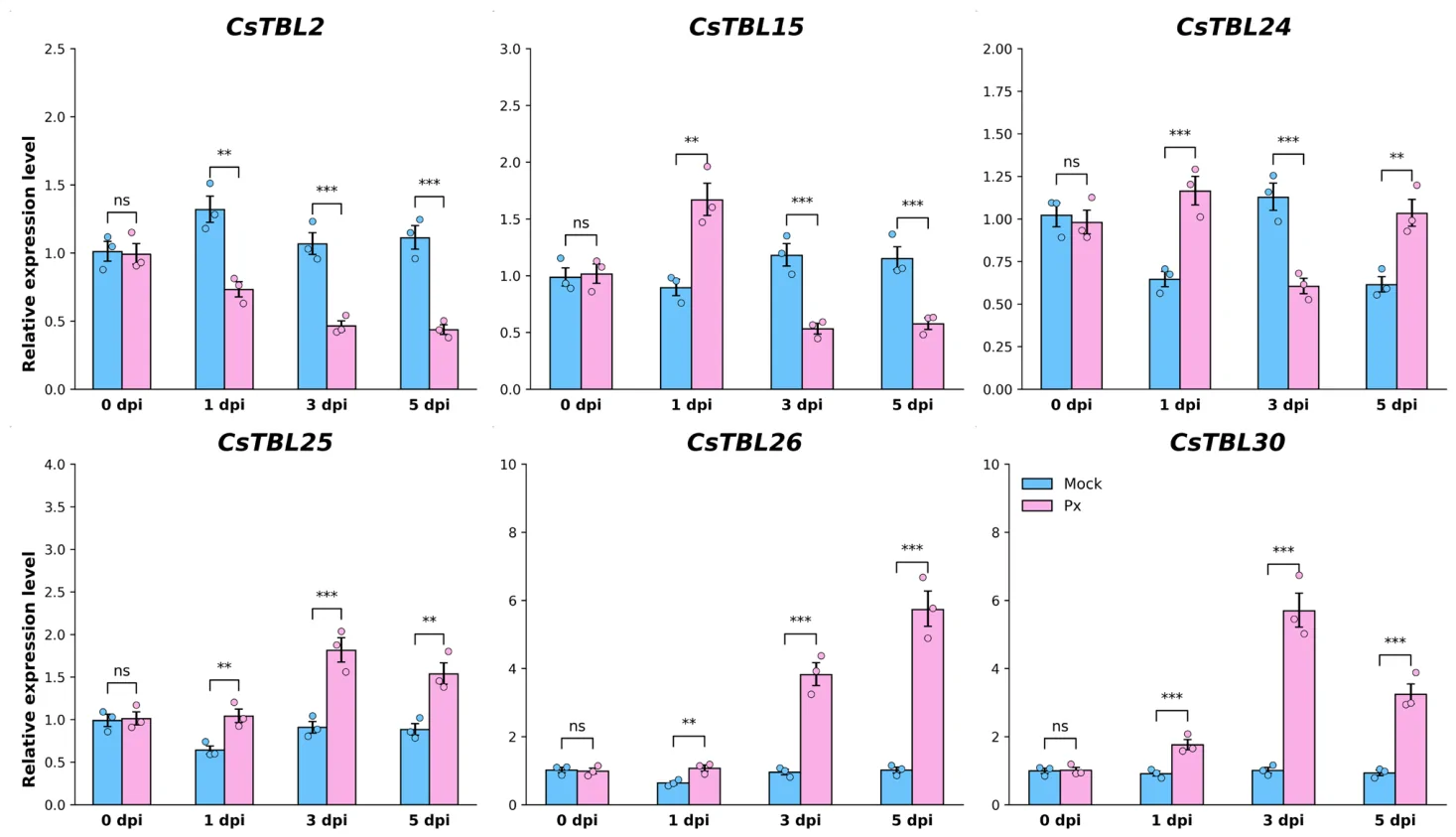
Dynamic expression patterns of selected *CsTBL* genes during *Podosphaera xanthii* infection. The expression profiles of *CsTBL2*, *CsTBL15*, *CsTBL24*, *CsTBL25*, *CsTBL26*, and *CsTBL30* were analyzed by RT-qPCR at 0, 1, 3, and 5 days post-inoculation (dpi). Cucumber seedlings were inoculated with *Podosphaera xanthii* (Px), and mock-inoculated plants were used as controls. Relative transcript levels were calculated using the 2^−ΔΔCt^ method with the average expression level of 0 dpi samples as the calibrator. Data are shown as mean ± SD from three biological replicates. Statistical significance was determined using two-way ANOVA followed by Tukey’s multiple-comparison test. Asterisks indicate significant differences between Px-inoculated and mock-inoculated plants at the same time point (** *p* < 0.01, *** *p* < 0.001).

RT-qPCR analysis revealed distinct temporal expression patterns among the six selected *CsTBL* genes following *P. xanthii* inoculation (Figure 11). Relative to the corresponding time-matched mock controls, *CsTBL2* showed significantly lower transcript abundance at 1, 3, and 5 dpi, indicating sustained post-inoculation repression. *CsTBL15* was significantly induced at 1 dpi but was significantly repressed at 3 and 5 dpi, representing a transient early-induction pattern. *CsTBL24* exhibited a biphasic response, with significant induction at 1 dpi, significant repression at 3 dpi, and renewed induction at 5 dpi.

*CsTBL25* showed significantly higher expression than the corresponding mock control at all three post-inoculation time points and had its highest observed mean expression level at 3 dpi. CsTBL26 was significantly induced at all three post-inoculation time points, and the magnitude of induction increased across the sampled time course, with the highest observed mean at 5 dpi. *CsTBL30* was significantly induced at 1, 3, and 5 dpi, reached its highest observed mean expression level at 3 dpi, and showed a lower observed mean at 5 dpi while remaining significantly higher than the corresponding mock control.

These results demonstrate that the selected *CsTBL* genes exhibit distinct temporal transcriptional responses following *P. xanthii* inoculation. However, changes in transcript abundance alone do not establish whether these genes positively or negatively regulate powdery mildew resistance.

## 4. Discussion

All 37 CsTBL proteins identified in this study retained the characteristic GDS and DxxH motifs of the TBL/DUF231 family and shared a broadly conserved core motif architecture, indicating that they possess the fundamental structural features associated with plant cell-wall polysaccharide O-acetylation. Structural and biochemical studies of *Arabidopsis* XOAT1 have demonstrated that the Ser residue within the GDS motif, together with the Asp and His residues in the DxxH region, forms the catalytic center responsible for xylan acetyl transfer. By contrast, other TBL members, such as AXY4 and AXY4L, act on xyloglucan, indicating that family members share a common catalytic framework but differ in substrate specificity [3,6]. The conservation of the GDS and DxxH-containing motifs is consistent with preservation of the characteristic catalytic framework of TBL proteins. However, whether the DCSH sequence context alters catalytic efficiency, substrate preference, or other biochemical properties cannot be determined from motif conservation alone and will require direct biochemical characterization. Nevertheless, the current sequence and structural predictions are insufficient to determine the authentic substrate of each member, and the entire *CsTBL* family should not be classified directly as xylan acetyltransferases.

The *CsTBL* family contained genes derived from dispersed, segmental, and tandem duplications and retained varying degrees of synteny with TBL genes in *Arabidopsis* and melon. These patterns indicate that the current family composition reflects both the retention of ancient genes and lineage-specific expansion within cucumber. The interspecific syntenic relationships should nevertheless be interpreted cautiously in terms of orthology. One-to-one syntenic pairs, particularly when also supported by close phylogenetic relationships, represent stronger candidates for orthologous relationships. By contrast, one-to-many and many-to-many patterns may reflect ancestral or lineage-specific duplication followed by differential retention; therefore, they may include both orthologous and paralogous relationships. This distinction is particularly relevant for cucumber, whose genome has undergone extensive chromosome fusion and rearrangement since divergence from melon. Such rearrangements can alter local genomic context and complicate simple one-to-one correspondence between homologous genes. Therefore, the synteny analysis is used here primarily to identify conserved genomic relationships and duplication history rather than to assign definitive orthology to every linked gene pair. Beyond the evolutionary relationships inferred from synteny, duplicated *CsTBL* genes also showed evidence of transcriptional divergence. For example, the tandemly duplicated genes *CsTBL24* and *CsTBL25* shared similar basal expression in several fruit tissues but displayed distinct temporal responses to powdery mildew inoculation. Likewise, although *CsTBL15* and *CsTBL26* showed a syntenic relationship, their post-inoculation expression patterns differed markedly: *CsTBL15* exhibited transient early induction followed by repression, whereas *CsTBL26* showed progressive induction during the middle and late stages. These contrasting expression patterns are consistent with transcriptional divergence among duplicated *CsTBL* genes. However, whether such transcriptional divergence is accompanied by functional divergence or reduced functional redundancy cannot be determined from expression data alone. The distinct combinations of hormone- and stress-responsive elements in their promoters are consistent with this interpretation. However, cis-regulatory element prediction provides only potential regulatory clues and cannot substitute for experimental validation of transcription factor binding or promoter activity.

GO enrichment analysis associated the *CsTBL* family predominantly with the Golgi apparatus, endomembrane system, cell-wall organization, polysaccharide metabolism, and O-acetyltransferase activity, consistent with the established roles of TBL proteins in modifying non-cellulosic polysaccharides within the Golgi apparatus. *CsTBL13*, *CsTBL15*, *CsTBL16*, *CsTBL17*, *CsTBL19*, and *CsTBL26* were annotated as putative xylan O-acetyltransferases, and several of these proteins clustered near functionally characterized Arabidopsis proteins involved in xylan acetylation. The agreement between phylogenetic placement and functional annotation provides mutually supportive evidence for candidate selection.

Tissue-expression analysis revealed distinct expression modules among *CsTBL* genes in roots, floral organs, ovaries, and different fruit tissue layers. Among the 37 family members, *CsTBL1*, *CsTBL7*, *CsTBL8*, *CsTBL17*, *CsTBL18*, *CsTBL21*, *CsTBL24*, *CsTBL25*, *CsTBL27*, and *CsTBL36* showed relatively high expression in ovary- or fruit-associated samples. The fruit samples analyzed here mainly represent anthesis and early post-pollination development. Accordingly, the observed expression patterns are more consistent with potential roles in early fruit growth, cell expansion, and tissue differentiation than with later ripening or softening. The distinct expression patterns observed among mesocarp, endocarp, and exocarp samples further suggest spatial specialization in fruit cell-wall remodeling.

However, expression enrichment alone does not establish a direct role in fruit-quality traits. In the absence of measurements of fruit firmness, texture, polysaccharide composition, or wall acetylation in *CsTBL* mutants, these genes should be regarded as candidates for future functional studies rather than as demonstrated regulators of fruit quality. In particular, *CsTBL24* and *CsTBL25* are of interest because they are expressed in fruit-associated tissues while also showing distinct transcriptional responses during powdery mildew infection.

Following powdery mildew inoculation, the six candidate *CsTBL* genes exhibited clearly differentiated temporal responses. *CsTBL2* was continuously repressed, *CsTBL15* showed early induction followed by a decline, and *CsTBL24* displayed intermediate-stage repression followed by a late rebound. By contrast, *CsTBL25*, *CsTBL26*, and *CsTBL30* were induced mainly during the middle and late stages of infection. These non-uniform patterns demonstrate that different *CsTBL* genes are transcriptionally responsive at different stages following powdery mildew inoculation rather than exhibiting a single coordinated expression pattern. Whether these distinct temporal expression profiles correspond to different functional roles in cell-wall regulation or infection responses remains to be experimentally determined. Previous studies have similarly shown that TBL-mediated cell-wall modification does not exert a uniformly positive or negative effect on infection response. The *Arabidopsis pmr5* mutant exhibits altered cell-wall composition and enhanced resistance to powdery mildew, and PMR5 was subsequently shown to possess pectin acetyltransferase activity, supporting a link between pectin acetylation and powdery mildew interactions [7,34]. In contrast, the rice *ostbl1* single mutant and *tbl1 tbl2* double mutant show reduced xylan acetylation and increased susceptibility to bacterial blight [8]. These contrasting phenotypes indicate that the effects of TBL-mediated polysaccharide acetylation on plant immunity may be jointly determined by the polysaccharide substrate, tissue type, pathogen species, and infection stage. Accordingly, increased or decreased expression in the present study indicates that the corresponding *CsTBL* genes respond to powdery mildew inoculation but does not establish whether they function as positive or negative regulators of infection response.

Candidate prioritization after RT-qPCR validation was based on the integration of functional annotation, phylogenetic context, tissue-expression patterns, and the magnitude and temporal consistency of the experimentally observed transcriptional responses rather than on any single criterion. *CsTBL26* carries a putative xylan O-acetyltransferase annotation and showed progressive induction during the middle and late stages following powdery mildew inoculation. These features make it an attractive candidate for future functional studies of the possible relationship between cell-wall polysaccharide acetylation and infection-associated transcriptional responses. *CsTBL15*, which exhibited transient early induction followed by sustained repression, may be useful for investigating early infection responses and their potential relationship with cell-wall regulation. *CsTBL24* and *CsTBL25* were expressed in fruit-associated tissues but showed distinct responses to powdery mildew, making them suitable candidates for examining tissue-dependent functions during fruit development and powdery mildew infection. *CsTBL2* provides a contrasting candidate characterized by sustained repression. *CsTBL30* showed significant post-inoculation induction and reached its highest observed expression at 3 dpi, making it an additional candidate for investigating powdery mildew-responsive transcription. Similar family diversification and stress-associated functions have been reported for *TBL* genes in rose and other crop species, suggesting that diversification of TBL family members and their association with stress responses may be broadly conserved across crops.

Future studies should combine subcellular localization, recombinant enzyme assays, substrate-specificity measurements, wall-bound acetate quantification, and polysaccharide structural analysis. These approaches should be integrated with assessments of fruit texture and powdery mildew infection phenotypes in CRISPR-generated mutants to establish direct causal relationships among *CsTBL* genes, cell-wall polysaccharide acetylation, fruit quality, and disease responses.

From an agricultural perspective, *CsTBL* genes showing fruit-associated expression or reproducible pathogen-responsive transcription provide candidate targets for future studies of fruit cell-wall properties and disease responses. Because polysaccharide acetylation can influence cell-wall architecture, experimentally validated CsTBL alleles may ultimately be useful for investigating traits related to fruit texture, post-harvest performance, or disease resistance. However, the present study does not establish direct effects on shelf life, fruit quality, or resistance, and these potential applications will require validation through genetic manipulation, cell-wall compositional analysis, and agronomic phenotyping.

## 5. Conclusions

In this study, 37 *CsTBL* genes were systematically identified in the cucumber genome. Analyses of sequence characteristics, gene duplication, and cross-species comparisons among Arabidopsis, cucumber, and melon revealed evolutionary diversification of the family around a conserved TBL/DUF231 structural framework. Functional annotation and expression analyses associated the *CsTBL* family primarily with cell-wall polysaccharide metabolism and O-acetylation. Individual family members exhibited distinct expression patterns across tissues, fruit developmental stages, and biotic and abiotic stress conditions.

RT-qPCR analysis following powdery mildew inoculation revealed distinct temporal transcriptional responses among the six selected *CsTBL* genes, highlighting several candidates for subsequent functional investigation. Among these genes, CsTBL26 was annotated as a putative xylan O-acetyltransferase and showed progressive post-inoculation induction, making it an attractive candidate for future functional studies. CsTBL24 and CsTBL25 showed fruit-associated expression together with distinct powdery mildew-responsive profiles, whereas CsTBL30 showed sustained post-inoculation induction. These expression and annotation patterns generate testable hypotheses for future studies but do not establish direct roles in fruit development or disease responses. This study provides an evolutionary and expression-based framework for the functional characterization of the cucumber TBL family. However, the polysaccharide substrates and enzymatic activities of individual CsTBL proteins, as well as their direct effects on fruit traits and infection responses, require further experimental validation.

## Figures and Tables

**Table 1:** Real-time quantitative PCR primers.

Gene	Forward Prime	Reverse Primer	Length (bp)
*CsTBL2*	CGGAACAGCGACCACCATAA	CTGCCAACGGTACTTGAGGT	195
*CsTBL15*	CTGTCGTCCTCTGTGCCTTT	TCTGGGTCAAACTCGAACCG	193
*CsTBL24*	CCATTGGTTCTTCCGTCCGA	ATGCGACGGTGCAAATGTTC	196
*CsTBL25*	TTGGAGACTCCGTCGCTAGA	AACCATTCTGGTCTGCGTCC	198
*CsTBL26*	CGGAACGAAACAGGTGGAGA	AAGATCGCATTCTTCGGGGG	187
*CsTBL30*	ATAGAGTCGGCAGAAGCGTG	GGTCCACCCATTTAGCCCAA	184
*CsActin*	CCCTCATGCCATTCTCCGTT	TGCTCTTTGCATCTCGAGTT	185

## Data Availability

The original contributions presented in this study are included in the article. Further inquiries can be directed to the corresponding authors.

## Supplementary Materials

The following supporting information can be downloaded at: https://www.mdpi.com/article/10.3390/biology15171454/s1, Figure S1: Stability of Ct values across experimental conditions. Figure S2: Distribution of predicted secondary structure elements in 37 CsTBL proteins. Figure S3: Predicted tertiary structures of CsTBL proteins. Figure S4: Dynamic expression patterns of the *CsTBL* gene family under different disease treatments. Figure S5: Dynamic expression patterns of the *CsTBL* gene family under cold, salt, and heat stress. Table S1: Amplification efficiencies of primer pairs used for RT-qPCR. Table S2: Two-way ANOVA results for the effects of inoculation treatment, sampling time, and their interaction on the expression of six *CsTBL* genes. Table S3: Physicochemical properties of cucumber *CsTBL* family members. Table S4: Predicted secondary structure composition of CsTBL proteins. Table S5: GDS and DxxH/DCSH motif variants in the 37 CsTBL proteins based on full-length multiple sequence alignment. Data S1: Full-length multiple sequence alignment of the 37 CsTBL proteins in aligned FASTA format.

## Abbreviations

The following abbreviations are used in this manuscript:

dpi	Days post-inoculation
CDS	Coding sequence
UTR	Untranslated region
GRAVY	Grand Average of Hydropathicity
CC	Cucumber scab
PM	Powdery mildew
GM	Gray mold
RD	Root differentiation zone
ART	Adventitious root tips
ALS	Bacterial angular leaf spot
FstLTB	First leaf blade
ALF	Adult leaf blade
SAM	Shoot apical meristem
PT_FF	Petal of female flower
GN_FF	Gynoecium of female flower
PT_MF	Petal of male flower
SA_MF	Stamen of male flower
OA_0DAF	Ovary at 0 days after flowering
MS_PF_10DAF	Mesocarp of pollinated fruit at 10 days after flowering
EN_PF_10DAF	Endocarp of pollinated fruit at 10 days after flowering
EX_PF_10DAF	Exocarp of pollinated fruit at 10 days after flowering

## References

[1] Potikha T., Delmer D.P. (1995). A mutant of Arabidopsis thaliana displaying altered patterns of cellulose deposition. Plant J..

[2] Volker B., Silvia N., Lutz N., Dana S., Aurélie U., Ravit E., Staffan P., Deborah D., Wolf-Rüdiger S. (2010). Trichome Birefringence and its homolog AT5G01360 encode plant-specific DUF231 proteins required for cellulose biosynthesis in Arabidopsis. Plant Physiol..

[3] Gille S., de Souza A., Xiong G., Benz M., Cheng K., Schultink A., Reca I.B., Pauly M. (2011). O-acetylation of Arabidopsis hemicellulose xyloglucan requires AXY4 or AXY4L, proteins with a TBL and DUF231 domain. Plant Cell.

[4] Ruiqin Z., Dongtao C., Zheng-Hua Y. (2018). Members of the DUF231 family are O-acetyltransferases catalyzing 2-O- and 3-O-acetylation of mannan. Plant Cell Physiol..

[5] Stranne M., Ren Y., Fimognari L., Birdseye D., Yan J., Bardor M., Mollet J.C., Komatsu T., Kikuchi J., Scheller H.V. (2018). TBL 10 is required for O-acetylation of pectic rhamnogalacturonan-I in Arabidopsis thaliana. Plant J..

[6] Lunin V.V., Wang H.-T., Bharadwaj V.S., Alahuhta M., Peña M.J., Yang J.-Y., Archer-Hartmann S.A., Azadi P., Himmel M.E., Moremen K.W. (2020). Molecular mechanism of polysaccharide acetylation by the Arabidopsis xylan O-acetyltransferase XOAT1. Plant Cell.

[7] Vogel J.P., Raab T.K., Somerville C.R., Somerville S.C. (2004). Mutations in PMR5 result in powdery mildew resistance and altered cell wall composition. Plant J..

[8] Gao Y., He C., Zhang D., Liu X., Xu Z., Tian Y., Liu X.-H., Zang S., Pauly M., Zhou Y. (2017). Two trichome birefringence-like proteins mediate xylan acetylation, which is essential for leaf blight resistance in rice. Plant Physiol..

[9] Tian Y., Zhang S., Liu X., Zhang Z. (2021). Global investigation of TBL gene family in rose (*Rosa chinensis*) unveils RcTBL16 is a susceptibility gene in gray mold resistance. Front. Plant Sci..

[10] Zhu X., Ma X., Hu W., Xing Y., Huang S., Chen Z., Fang L. (2024). Genome-wide identification of TBL gene family and functional analysis of GhTBL84 under cold stress in cotton. Front. Plant Sci..

[11] Huang S., Li R., Zhang Z., Li L., Gu X., Fan W., Lucas W.J., Wang X., Xie B., Ni P. (2009). The genome of the cucumber, *Cucumis sativus* L. Nat. Genet..

[12] Ando K., Carr K.M., Grumet R. (2012). Transcriptome analyses of early cucumber fruit growth identifies distinct gene modules associated with phases of development. BMC Genom..

[13] Li D., Cuevas H.E., Yang L., Li Y., Garcia-Mas J., Zalapa J., Staub J.E., Luan F., Reddy U., He X. (2011). Syntenic relationships between cucumber (*Cucumis sativus* L.) and melon (*C. melo* L.) chromosomes as revealed by comparative genetic mapping. Bmc Genom..

[14] Benjak A., Lowy E., Alioto T., Capella-Gutiérrez S., Blanca J., Cañizares J., Ziarsolo P., Gonzalez-Ibeas D., Rodríguez-Moreno L., Droege M. (2012). The genome of melon (*Cucumis melo* L.). Proc. Natl. Acad. Sci. USA.

[15] Guan J., Miao H., Zhang Z., Dong S., Zhou Q., Liu X., Beckles D.M., Gu X., Huang S., Zhang S. (2024). A near-complete cucumber reference genome assembly and Cucumber-DB, a multi-omics database. Mol. Plant.

[16] Berardini T.Z., Reiser L., Li D., Mezheritsky Y., Muller R., Strait E., Huala E. (2015). The Arabidopsis information resource: Making and mining the “gold standard” annotated reference plant genome. Genesis.

[17] Chen C., Wu Y., Li J., Wang X., Zeng Z., Xu J., Liu Y., Feng J., Chen H., He Y. (2023). TBtools-II: A “one for all, all for one” bioinformatics platform for biological big-data mining. Mol. Plant.

[18] Zhao Y., Jing H., Zhao P., Chen W., Li X., Sang X., Lu J., Wang H. (2021). Gh TBL34 is associated with verticillium wilt resistance in cotton. Int. J. Mol. Sci..

[19] Li W., Godzik A. (2006). Cd-hit: A fast program for clustering and comparing large sets of protein or nucleotide sequences. Bioinform..

[20] Guruprasad K., Reddy B.B., Pandit M.W. (1990). Correlation between stability of a protein and its dipeptide composition: A novel approach for predicting in vivo stability of a protein from its primary sequence. Protein Eng. Des. Sel..

[21] Kyte J., Doolittle R.F. (1982). A simple method for displaying the hydropathic character of a protein. J. Mol. Biol..

[22] Tamura K., Stecher G., Kumar S. (2021). MEGA11: Molecular evolutionary genetics analysis version 11. Mol. Biol. Evol..

[23] Letunic I., Bork P. (2007). Interactive Tree of Life: An online tool for phylogenetic tree display and annotation. Bioinformatics.

[24] Bailey T.L., Johnson J., Grant C.E., Noble W.S. (2015). The MEME suite. Nucleic Acids Res..

[25] Lescot M., Déhais P., Thijs G., Marchal K., Moreau Y., Van de Peer Y., Rouzé P., Rombauts S. (2002). PlantCARE, a database of plant cis-acting regulatory elements and a portal to tools for in silico analysis of promoter sequences. Nucleic Acids Res..

[26] Geourjon C., Deleage G. (1995). SOPMA: Significant improvements in protein secondary structure prediction by consensus prediction from multiple alignments. Bioinformatics.

[27] Waterhouse A., Bertoni M., Bienert S., Studer G., Tauriello G., Gumienny R., Heer F.T., de Beer T.A.P., Rempfer C., Bordoli L. (2018). SWISS-MODEL: Homology modelling of protein structures and complexes. Nucleic Acids Res..

[28] Wang Y., Tang H., DeBarry J.D., Tan X., Li J., Wang X., Lee T.-h., Jin H., Marler B., Guo H. (2012). MCScanX: A toolkit for detection and evolutionary analysis of gene synteny and collinearity. Nucleic Acids Res..

[29] Conesa A., Götz S., García-Gómez J.M., Terol J., Talón M., Robles M. (2005). Blast2GO: A universal tool for annotation, visualization and analysis in functional genomics research. Bioinformatics.

[30] Carbon S., Douglass E., Good B.M., Unni D.R., Harris N.L., Mungall C.J., Basu S., Chisholm R.L., Dodson R.J., Hartline E. (2020). The Gene Ontology resource: Enriching a GOld mine. Nucleic Acids Res..

[31] Takamatsu S., Havrylenko M., Wolcan S.M., Matsuda S., Niinomi S. (2008). Molecular phylogeny and evolution of the genus Neoerysiphe (Erysiphaceae, Ascomycota). Mycol. Res..

[32] Wan H., Zhao Z., Qian C., Sui Y., Malik A.A., Chen J. (2010). Selection of appropriate reference genes for gene expression studies by quantitative real-time polymerase chain reaction in cucumber. Anal. Biochem..

[33] Livak K.J., Schmittgen T.D. (2001). Analysis of relative gene expression data using real-time quantitative PCR and the 2−ΔΔCT method. Methods.

[34] Chiniquy D., Underwood W., Corwin J., Ryan A., Szemenyei H., Lim C.C., Stonebloom S.H., Birdseye D.S., Vogel J., Kliebenstein D. (2019). PMR 5, an acetylation protein at the intersection of pectin biosynthesis and defense against fungal pathogens. Plant J..

