# Supplementary material for: Genome-Wide Identification and Characterization of the TBL Gene Family and Temporal Expression Dynamics During Powdery Mildew Infection in Cucumber (Cucumis sativus)
Source: Biology (Basel). 2026 Aug 25;15(17):1454. doi: 10.3390/biology15171454 (PMC13565612; doi:10.3390/biology15171454)
Supplement: Supplementary file 1 [file biology-15-01454-s001.zip › Supplementary Document.pdf]

Supplementary Table S1. Amplification efficiencies of primer pairs used for RT-qPCR.

Primer pair	Efficiency (%)
CsActin	99.2
CsTBL2	102.1
CsTBL15	96.8
CsTBL24	103.2
CsTBL25	98.4
CsTBL26	100.5
CsTBL30	95.8

Supplementary Table S2. Two-way ANOVA results for the effects of inoculation treatment, sampling time, and their interaction on the expression of six *CsTBL* genes.

Gene	Treatment	P	Time	P	Treatment×Time	P
CsTBL2	F(1,16)=114.680	1.05×10^{-8}	F(3,16)=13.126	1.40×10^{-4}	F(3,16) =13.335	1.28×10^{-4}
CsTBL15	F(1,16)=11.512	3.72×10^{-3}	F(3,16)=10.700	4.20×10^{-4}	F(3,16) =28.368	1.22×10^{-6}
CsTBL24	F(1,16)=5.011	3.98×10^{-2}	F(3,16)=4.102	2.45×10^{-2}	F(3,16) =32.154	5.24×10^{-7}
CsTBL25	F(1,16)=70.751	2.88×10^{-7}	F(3,16)=14.220	8.90×10^{-5}	F(3,16) =7.643	2.16×10^{-3}
CsTBL26	F(1,16)=242.732	4.32×10^{-11}	F(3,16)=78.144	9.00×10^{-10}	F(3,16) =48.102	3.14×10^{-8}
CsTBL30	F(1,16)=233.295	5.82×10^{-11}	F(3,16)=40.339	1.09×10^{-7}	F(3,16) =37.522	1.81×10^{-7}

Supplementary Table S3. Physicochemical properties of cucumber CsTBL family members

Name	Sequence ID	Number of Amino Acid(aa)	Molecular Weight(Da)	Theoretical pI	Instability Index	Aliphatic Index	Grand Average of Hydropathicity
CsTBL1	CsaV4_1G002742.mRNA6	373	42291.39	8.32	46.25	81.53	-0.24
CsTBL2	CsaV4_1G002929.mRNA4	390	44756.65	5.89	33.68	68.54	-0.395
CsTBL3	CsaV4_2G000010.mRNA5	613	69374.86	6.39	46.19	81.52	-0.458
CsTBL4	CsaV4_2G000687.mRNA3	479	54458.43	8.38	42.69	72.05	-0.456
CsTBL5	CsaV4_2G001641.mRNA3	428	48941.84	8.73	49.96	84.58	-0.191
CsTBL6	CsaV4_2G002494.mRNA1	553	63815.42	7.29	49.22	62.89	-0.666
CsTBL7	CsaV4_3G002130.mRNA2	448	51441.97	8.21	42.77	78.88	-0.31
CsTBL8	CsaV4_3G003822.mRNA1	613	69814.62	8.71	46.84	61.14	-0.688
CsTBL9	CsaV4_3G004182.mRNA1	473	54730.09	8.46	37.06	73.38	-0.294
CsTBL10	CsaV4_4G000127.mRNA1	530	61158.44	9.32	33.3	72.02	-0.548
CsTBL11	CsaV4_4G000842.mRNA2	371	42508.22	6.51	26.39	76.66	-0.369
CsTBL12	CsaV4_4G000843.mRNA2	351	39724.69	7.57	35.36	89.6	-0.109
CsTBL13	CsaV4_4G000851.mRNA3	415	48392.35	7.24	35.97	84.02	-0.412
CsTBL14	CsaV4_4G001222.mRNA11	403	46407.74	8.84	44.42	69.45	-0.44
CsTBL15	CsaV4_4G002175.mRNA2	440	50940.53	8.94	39.53	72.41	-0.392
CsTBL16	CsaV4_4G002201.mRNA2	424	48945.87	8.49	44.96	78.18	-0.452
CsTBL17	CsaV4_4G002202.mRNA10	445	52236.44	6.97	52.15	77.3	-0.549
CsTBL18	CsaV4_5G000211.mRNA9	457	52619.63	6.56	38.2	84.07	-0.421
CsTBL19	CsaV4_5G000866.mRNA1	429	49868.88	6.05	49.99	72.94	-0.436
CsTBL20	CsaV4_5G001864.mRNA2	363	41762.48	5.94	34.37	77.05	-0.382
CsTBL21	CsaV4_5G002621.mRNA2	444	51003.22	6.68	48.76	80.16	-0.383
CsTBL22	CsaV4_5G002936.mRNA1	624	69445.12	6.36	41.36	70.75	-0.514
CsTBL23	CsaV4_5G002983.mRNA1	378	44325.28	7.65	58.51	68.84	-0.532
CsTBL24	CsaV4_5G003373.mRNA6	441	51030.91	6.54	38.24	71.63	-0.463
CsTBL25	CsaV4_5G003374.mRNA1	429	50245.37	7.54	37.46	70.44	-0.398
CsTBL26	CsaV4_6G000196.mRNA1	508	58892.43	5.04	52.94	74.59	-0.556
CsTBL27	CsaV4_6G001328.mRNA5	441	51105.52	8.67	51.89	69.57	-0.368
CsTBL28	CsaV4_6G001662.mRNA2	371	42545.55	8.91	32.92	72.99	-0.275
CsTBL29	CsaV4_6G002568.mRNA1	419	48990.02	8.47	45.33	74.44	-0.446
CsTBL30	CsaV4_6G002602.mRNA1	358	40734.77	5.88	38.75	85.22	-0.18
CsTBL31	CsaV4_6G002907.mRNA2	444	52295.89	6.57	50.44	59.86	-0.631
CsTBL32	CsaV4_6G002943.mRNA1	510	57560.84	9.13	37.53	74.31	-0.477
CsTBL33	CsaV4_6G003919.mRNA11	362	41730	9.14	41.35	79.64	-0.331
CsTBL34	CsaV4_7G000690.mRNA5	*400*	*46432.53*	*8.78*	*47.8*	*88.47*	*-0.313*
CsTBL35	CsaV4_7G001596.mRNA1	*425*	*49224.19*	*6.8*	*42.12*	*78.82*	*-0.372*
CsTBL36	CsaV4_7G001617.mRNA3	*418*	*48537.21*	*5.98*	*44.23*	*82.54*	*-0.285*
CsTBL37	CsaV4_7G002266.mRNA7	*467*	*53422.78*	*8.46*	*50.56*	*73.04*	*-0.508*

Supplementary Table S4. Predicted secondary structure composition of CsTBL proteins

Name	Alpha Helix(%)	Extended Strand(%)	Beta Turn(%)	Random coil(%)
CsTBL1	26.27	18.5	3.75	51.47
CsTBL2	25.9	16.15	3.33	54.62
CsTBL3	18.6	13.38	2.45	65.58
CsTBL4	18.58	12.53	3.13	65.76
CsTBL5	27.34	14.95	2.57	55.14
CsTBL6	18.08	13.2	3.8	64.92
CsTBL7	25	14.73	3.35	56.92
CsTBL8	13.7	11.42	3.26	71.62
CsTBL9	26.43	17.34	3.38	52.85
CsTBL10	16.98	12.64	2.83	67.55
CsTBL11	23.45	19.14	3.77	53.64
CsTBL12	25.36	17.38	4.56	52.71
CsTBL13	26.51	15.18	4.1	54.22
CsTBL14	27.54	15.14	4.22	53.1
CsTBL15	22.73	15.45	3.18	58.64
CsTBL16	25.94	15.33	4.72	54.01
CsTBL17	22.7	14.38	2.47	60.45
CsTBL18	22.98	15.75	3.5	57.77
CsTBL19	22.14	16.08	3.5	58.28
CsTBL20	24.24	20.66	4.96	50.14
CsTBL21	26.35	15.77	3.6	54.28
CsTBL22	19.39	11.54	2.4	66.67
CsTBL23	23.28	16.67	2.38	57.67
CsTBL24	24.72	15.19	3.4	56.69
CsTBL25	20.75	16.32	2.56	60.37
CsTBL26	21.65	15.55	4.72	58.07
CsTBL27	27.44	14.06	3.4	55.1
CsTBL28	25.61	16.44	4.04	53.91
CsTBL29	23.39	15.99	3.58	57.04
CsTBL30	29.05	17.04	4.19	49.72
CsTBL31	26.13	16.22	3.15	54.5
CsTBL32	18.04	15.1	2.94	63.92
CsTBL33	27.9	17.68	3.04	51.38
CsTBL34	26.75	16.25	4.5	52.5
CsTBL35	26.12	14.59	4	55.29
CsTBL36	27.27	15.07	3.59	54.07
CsTBL37	21.2	14.13	3.64	61.03

Supplementary Table S5. GDS and DxxH/DCSH motif variants in the 37 CsTBL proteins based on full-length multiple sequence alignment

ID	Protein length (aa)	GDS-like motif	GDS-like motif start (aa)	GDS-region sequence context	DxxH motif variant	DxxH motif start (aa)	DxxH-region sequence context
CsTBL1	373	GDS	126	GKKIMFVGDSLSLNMWE	DCSH	349	NGEHSGTDCSHWCLPGLP
CsTBL2	390	GDS	127	GRTVMFVGDSLGRNQWE	DCSH	367	ANPDRSADCSHWCLPGLP
CsTBL3	613	GDS	349	DKTLAFVGDSLGRQQFQ	DCLH	592	TAKVGVQDCLHWCLPGVP
CsTBL4	479	GDS	205	GKRLMLVGDSMNRNQFE	DCSH	448	KVSTRRQDCSHWCLPGVP
CsTBL5	428	GDS	166	SKRVVFVGDSMSRSQWE	DCSH	398	SNNPSVPDCSHWCLPGVP
CsTBL6	553	GDS	278	GRSLVFVGDSLNRNMWE	DCSH	522	RAGLRVQDCSHWCLPGVP
CsTBL7	448	GDN	162	HKAFAFVGDNIARNHVQ	DCIH	404	GNENLNNDCIHWCLPGPI
CsTBL8	613	GDS	337	GKRLVFVGDSLNRNMWE	DCSH	582	KSPLRFQDCSHWCLPGVP
CsTBL9	473	GDS	199	GKKLAFIGDSVARNHME	DCVH	445	KWMKGYNDCVHWCLPGPI
CsTBL10	530	GDS	262	GKRLVFVGDSINRNQWE	DCSH	502	KFSRGGEDCSHWCLPGVP
CsTBL11	371	GDS	120	GKKIMVVGDSLSNNMWQ	DCSH	347	NNSTPLLDCSHWCLAGVP
CsTBL12	351	GDS	99	GKSILFVGDSLSRNQWQ	DCSH	326	LGGRVGLDCSHWCLAGVP
CsTBL13	415	GDS	144	DKRIMFVGDSIQRGQFE	DCIH	386	SDPKSFADCIHWCLPGVP
CsTBL14	403	GDS	144	NTNIGFVGDSLNRNMFV	DCMH	377	AGGKKHDDCMHWCLPGLT
CsTBL15	440	GDS	172	GKRLMFVGDSLQRGQWQ	DCIH	419	ADPLHHADCIHWCLPGVP
CsTBL16	424	GDS	146	NKRMVFVGDSLNRGQWV	DCIH	395	SNPSSYADCIHWCLPGVP
CsTBL17	445	GDS	181	GKRLMFVGDSLNRGQWI	DCIH	424	SNPASYSDCIHWCLPGVP
CsTBL18	457	GDS	183	DKTWALIGDSISRNHVQ	DCLH	433	KNAKVQKDCLHWCLPGPI
CsTBL19	429	GDS	158	GKRMMFVGDSLNRGQFV	DCVH	404	ANPVSYADCVHWCLPGLQ
CsTBL20	363	GDS	108	GKKIMFIGDSISINQWE	DCTH	332	YNGFRGMDCTHWCIAGLP
CsTBL21	444	GDS	156	NKHIAFIGDSMARNQVE	DCVH	410	VGDRIQNDCVHWCLPGPV
CsTBL22	624	GDS	295	GKTLAFIGDSVARNQME	DCLH	548	DGKPPPQDCLHWCMPGPV
CsTBL23	378	GDS	128	SKRIMLVGDSIMRNQWE	DCSH	357	HQESLSSDCSHWCLPGVP
CsTBL24	441	GDS	158	DKSLAFVGDSVGRNQMQ	DCVH	405	ENVTLYNDCVHWCLPGPI
CsTBL25	429	GDS	154	GKSLAFVGDSVARNHMQ	DCVH	400	PRNGNIADCVHWCLPGPI
CsTBL26	508	GDS	235	GKRLMFVGDSLNRNQWE	DCIH	483	ADPSTYADCIHWCLPGLP
CsTBL27	441	GDS	166	NKRVVFVGDSIGRNQWE	DCSH	415	PAPPHRQDCSHWCLPGVP
CsTBL28	371	GDS	125	GKKIMFVGDSLSLNQWE	DCSH	348	SGEHSGTDCSHWCLPGLP

CsTBL29	419	GDS	159	GKRVIIVGDSLNRNQWE	DCSH	393	RKEEIIADCSHWCLPGLP
CsTBL30	358	GDS	110	GKSIMFVGDSLSLNQWQ	DCSH	333	FGGHSGMDCSHWCLAGVP
CsTBL31	444	GDS	161	NGRIVFVGDSIGRNQWE	DCSH	416	TPDDAPQDCSHWCLPGVP
CsTBL32	510	GDS	245	DKTIAFIGDSLGRQQFQ	DCLH	489	KGTSSGSDCLHWCLPGIP
CsTBL33	362	GDS	117	GKKIMFVGDSLSLNMWQ	DCSH	339	YSGEHGRDCSHWCLPGLP
CsTBL34	400	GDS	123	NRNIGFVGDSLNENFLT	DCMH	372	AVAVWGQDCMHWCLPGVP
CsTBL35	425	GDS	161	GKRVVFVGDSLSRTQWE	DCGH	395	GDNPSVQDCGHWCLPGFP
CsTBL36	418	GDS	142	NKSWAFIGDSIQRNHVQ	DCLH	391	GNGKVQTDCLHWCLPGPI
CsTBL37	467	GDS	201	NKTLAFIGDSLGRQQFQ	DCLH	446	RAILNTSDCLHWCLPGIP

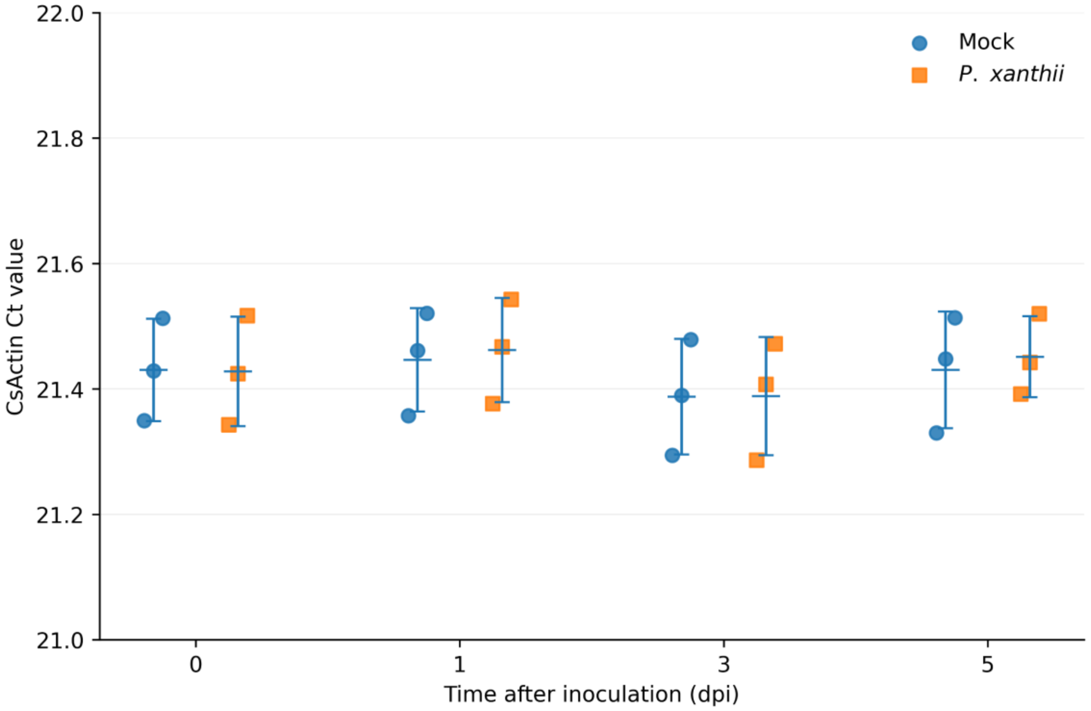

Supplementary Figure S1. Stability of Ct values across experimental conditions. *CsActin* Ct values were examined in mock-treated and *Podosphaera xanthii*-inoculated cucumber leaves at 0, 1, 3, and 5 days post inoculation (dpi). Each point represents one independent biological replicate and corresponds to the mean Ct value of three technical replicates (n = 3 biological replicates per treatment × time combination). Error bars indicate mean ± SD. Samples at 0 dpi were collected before treatment from plant groups subsequently designated for mock or *P. xanthii* treatment. The limited variation in *CsActin* Ct values across conditions supports its suitability as the reference gene for normalization under the experimental conditions used in this study.

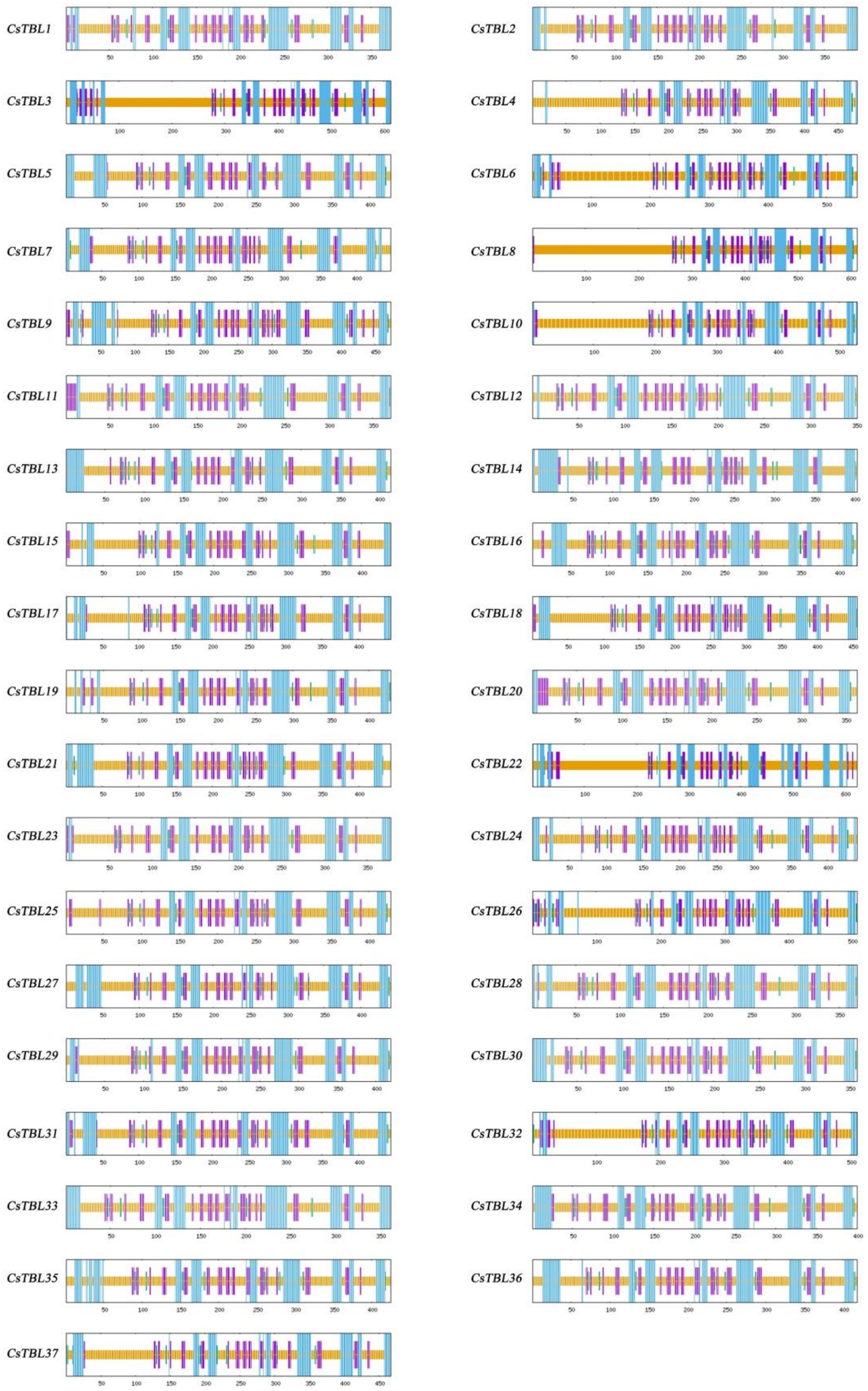

Supplementary Figure S2. Distribution of predicted secondary structure elements in 37 CsTBL proteins. The α-helices, extended strands, β-turns, and random coils of the 37 CsTBL proteins were predicted. Each panel represents one CsTBL protein, and different colors indicate different types of secondary structure elements. The horizontal axis represents amino acid residue positions, and the positions and lengths of the colored segments indicate the distribution ranges of the corresponding secondary structure elements along each protein sequence.

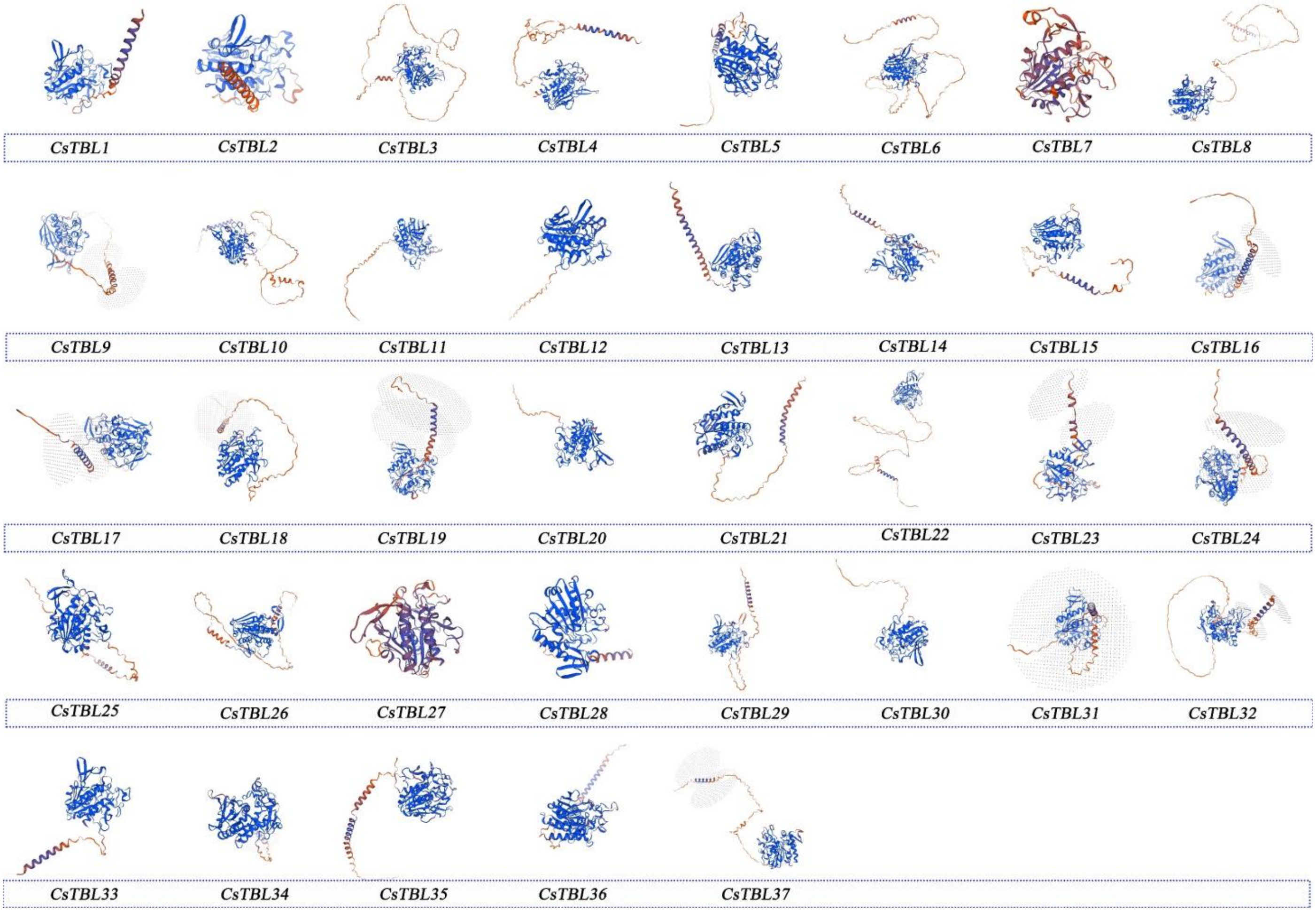

Supplementary Figure S3. Predicted tertiary structures of CsTBL proteins. The predicted three-dimensional structures of the 37 CsTBL proteins are shown, with the corresponding protein names indicated below each model. The models were visualized according to their predicted structures, showing the folded core, helical regions, and peripheral extensions. For figure arrangement and comparison among protein models, some structures were spatially rotated. This adjustment altered only the viewing orientation and did not affect the predicted structural information.

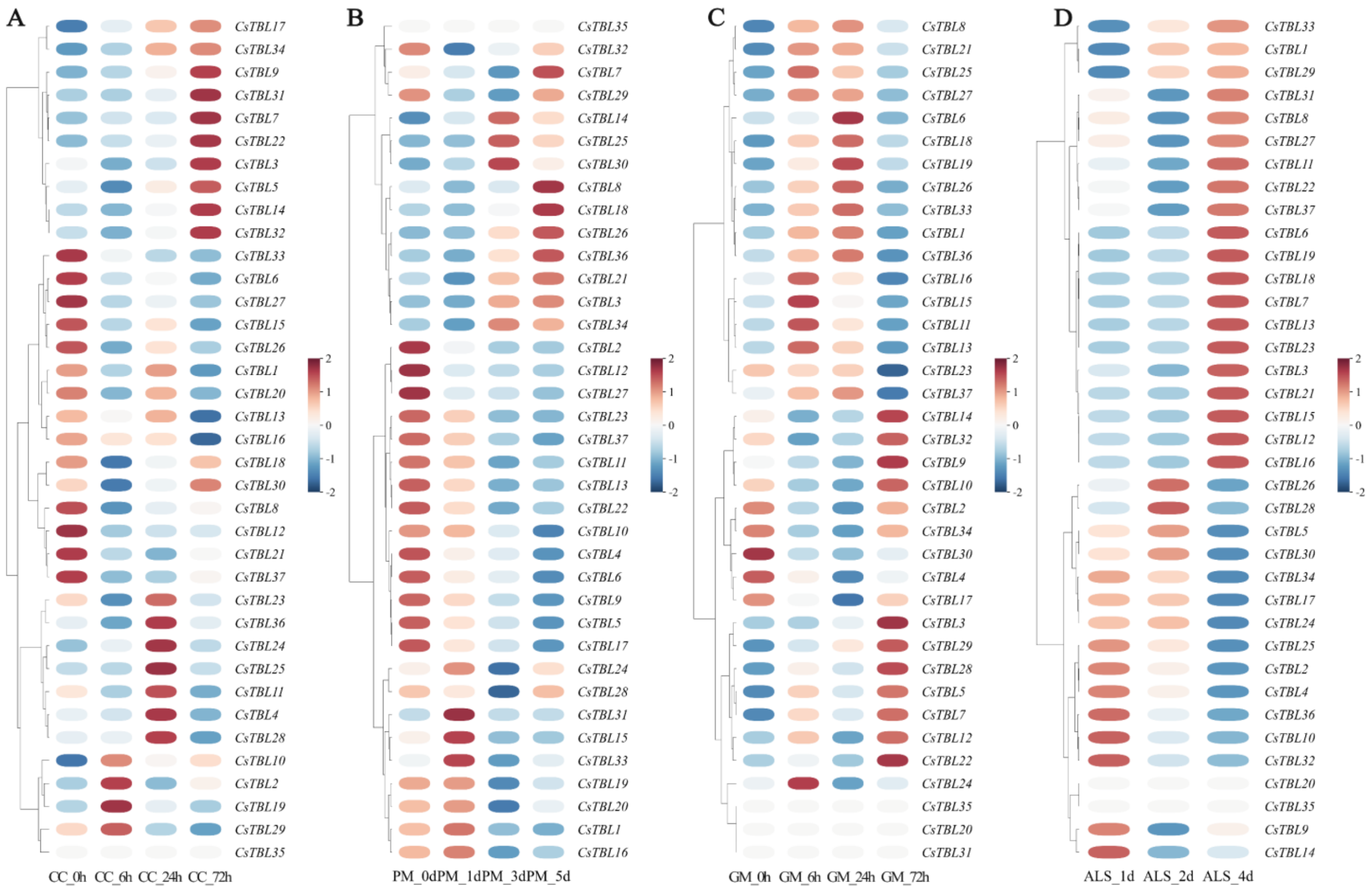

Supplementary Figure S4. Dynamic expression patterns of the *CsTBL* gene family under different disease treatments.(A) Cucumber scab (CC) at 0, 6, 24, and 72 h after treatment; (B) powdery mildew (PM) at 0, 1, 3, and 5 d after treatment; (C) gray mold (GM) at 0, 6, 12, and 48 h after treatment; and (D) angular leaf spot (ALS) at 1, 2, and 4 d after treatment. Colors represent relative gene expression levels, with red indicating relatively high expression and blue indicating relatively low expression. The clustering branches in different panels are independent of one another, and the colors are intended only to compare the relative expression changes of the same gene across the time course within each treatment.

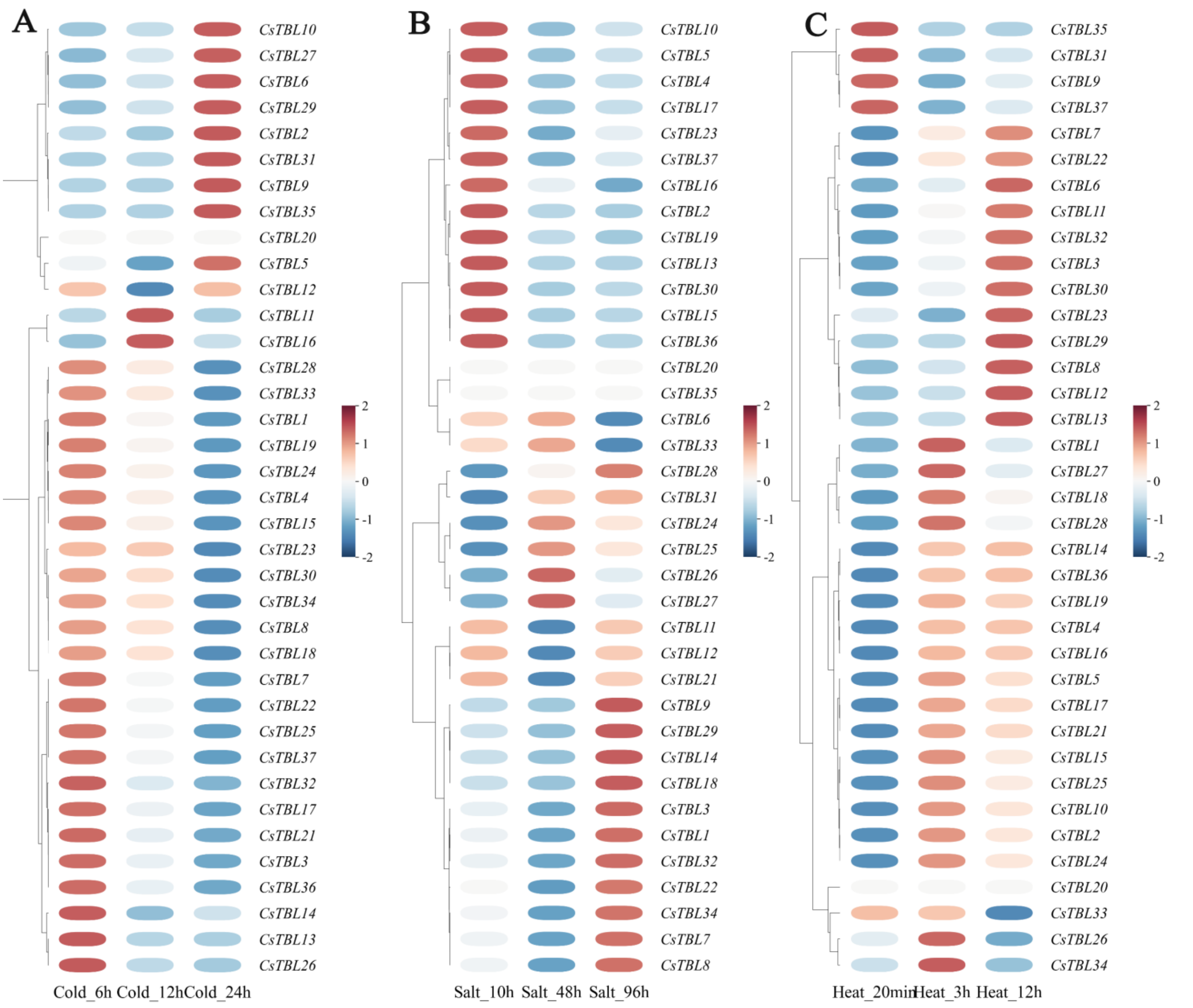

Supplementary Figure S5. Dynamic expression patterns of the *CsTBL* gene family under cold, salt, and heat stress.(A) Cold stress at 6, 12, and 24 h after treatment; (B) salt stress at 10, 48, and 96 h after treatment; and (C) heat stress at 20 min, 3 h, and 12 h after treatment. Red and blue indicate relatively high and relatively low expression levels, respectively, of the same gene across different treatment time points.
